# Genome-wide identification and analysis of the WRKY gene family and low-temperature stress response in *Prunus sibirica*

**DOI:** 10.1186/s12864-023-09469-0

**Published:** 2023-06-27

**Authors:** Quangang Liu, Shipeng Wang, Jiaxing Wen, Jianhua Chen, Yongqiang Sun, Shengjun Dong

**Affiliations:** 1 College of Forestry, Shenyang Agricultural University, Shenyang, China; 2 Key Laboratory for Silviculture of Liaoning Province, Shenyang Agricultural University, Shenyang, China

**Keywords:** Genome-wide identification, *WRKY* transcription factor, *Prunus sibirica*, Expression patterns, Low-temperature stress

## Abstract

**Background:**

*WRKY* transcription factors are a prominent gene family in plants, playing a crucial role in various biological processes including development, metabolism, defense, differentiation, and stress response. Although the *WRKY* gene family has been extensively studied and analysed in numerous plant species, research on *Prunus sibirica*’s *WRKY* genes (*PsWRKY*) remains lacking.

**Results:**

This study analysed the basic physicochemical properties, phylogeny, gene structure, cis-acting elements, and Gene ontology (GO) annotation of *PsWRKY* gene family members using bioinformatics methods based on the whole-genome data of *P. sibirica*. In total, 55 *WRKYs* were identified in *P. sibirica* and were heterogeneously distributed on eight chromosomes. Based on the phylogenetic analysis, these *WRKYs* were classified into three major groups: Group I, Group II (II-a, II-b, II-c, II-d, II-e), and Group III. Members of different subfamilies have different cis-acting elements, conserved motifs, and intron-exon structures, indicating functional heterogeneity of the *WRKY* family. Prediction of subcellular localisation indicated that *PsWRKYs* were mainly located in the nucleus. Twenty pairs of duplicated genes were identified, and segmental duplication events may play an important role in *PsWRKY* gene family expansion. Analysis of the Ka/Ks ratio showed that the *PsWRKY* family’s homologous genes were primarily purified by selection. Additionally, GO annotation analysis showed that the *WRKY* gene family was mainly involved in responses to stimuli, immune system processes, and reproductive processes. Furthermore, quantitative real-time PCR (qRT-PCR) analysis showed that 23 *PsWRKYs* were highly expressed in one or more tissues (pistils and roots) and *PsWRKYs* showed specific expression patterns under different low-temperature stress conditions.

**Conclusions:**

Our results provide a scientific basis for the further exploration and functional validation of *WRKYs* in *P. sibirica*.

**Supplementary Information:**

The online version contains supplementary material available at 10.1186/s12864-023-09469-0.

## Background

*WRKY* transcription factors are an essential family of transcriptional regulators in plants, with highly conserved protein structural domains [1–3]. The WRKY structural domain is composed of nearly 60 amino acid residues with an N-terminal DNA-binding activity-related WRKYGQK conserved structural domain (forming a β-strand). Additionally, the C-terminus contains zinc finger C_2_H_2_ (Cx_4 − 5_Cx_22 − 23_HxH) or C_2_HC (Cx_7_Cx_23_HxC) structural motifs that are involved in zinc finger protein interactions [3–5]. *WRKY* transcription factors are classified into three groups (I, II, and III) based on the number of conserved WRKY structural domains and zinc finger types [5, 6]. Group I contains two WRKY structural domains and the Cx_4 − 5_Cx_22 − 23_HxH zinc-finger motif; Group II contains one WRKY structural domain and the Cx_4 − 5_Cx_22 − 23_HxH zinc-finger motifs, which can be further divided into five subgroups (II a–e) depending on their phylogenetic branching and assembly [7, 8]; and Group III contains one WRKY structural domain and the Cx_7_Cx_23_HxC zinc-finger motifs [3].

The WRKY family has several specific biological functions owing to their distinctive structural domain. Numerous studies have demonstrated that *WRKY* transcription factors perform crucial regulatory roles in various aspects of plant biology, including growth and development, physiological and biochemical processes, as well as responses to biotic and abiotic stress [9–12]. For example, the overexpression of *MuWRKY3* enhances drought resistance in transgenic peanuts [13], whereas the overexpression of *GhWRKY34* increases the resistance of transgenic Arabidopsis leaves and roots to salt stress by enhancing the ability of plants to selectively absorb Na^+^ and K^+^ and maintain lower Na^+^/K^+^ levels [14]. In addition, the regulatory functions of *WRKYs* are closely related to several phytohormone-mediated signalling pathways. *VqWRKY31* enhances resistance to powdery mildew in grapevine through activation of salicylic acid defense signalling as well as promotion of the synthesis of specific disease-resistant metabolites [15]. Cold tolerance is a plant response to abiotic stress, and the gene expression levels of plants are differentially altered under low-temperature stress. Numerous studies have shown that the *WRKY* gene family plays a crucial role in regulating the response to cold stress; for example, in *Prunus mume*, overexpression of *PmWRKY57* increases cold resistance in transgenic Arabidopsis by reducing malondialdehyde content, increasing peroxidase and superoxide dismutase activity, proline content, and upregulating cold-responsive genes [16]. Notably, the tolerance of rice to low-temperature stress was enhanced by the upregulation of *OsWRKY71* expression under these conditions [17]. In *Acer truncatum*, 13 genes, including *AtruWRKY12*, *AtruWRKY13*, *AtruWRKY15*, and *AtruWRKY17*, were overexpressed from 0 to 12 h after low-temperature (4 °C) stress [18].

*Prunus sibirica* is a deciduous shrub belonging to the Rosaceae family, with a natural distribution concentrated in Eastern Siberia, Eastern and Southeastern Mongolia, and Northern and Northeastern China [19]. *P. sibirica* is an excellent tree species that combines ecological and economic benefits and can grow normally in poor conditions including drought, infertile soil, cold, and sandy winds. One of the most important tree species for the afforestation of barren mountains that can contribute to improving the environment by controlling various factors such as wind, sand, and water conservation [20]. *P. sibirica* seeds have rich economic value and are used in many areas including food, medicine, and industry [21]. However, frost damage during flowering limits the development of the *P. sibirica* industry. *P. sibirica* is an early spring-flowering plant that is susceptible to severe cold and frost during flowering, resulting in flower and fruit damage, reduced fruit yield, and even crop failure, leading to huge losses in production [22]. Therefore, mining and identification of potential genes related to frost resistance in the flowering organs are important for the development of frost-resistant and productive varieties of *P. sibirica*. Recent studies on *P. sibirica* have primarily concentrated on its molecular markers, genetic diversity, and almond composition [23–28]. However, there is a scarcity of research on its gene family. The publication of the complete genome sequence of *P. sibirica* is a significant milestone in molecular biology research and is of vital importance to further study the regulatory mechanisms of genes related to low-temperature stress, collecting cold resistance gene resources, using molecular breeding to enhance the cold resistance of *P. sibirica* and other forest tree species, and increase fruit production and quality.

In the present study, the members of the *PsWRKY* gene family were identified based on genome-wide data from *P. sibirica*, and comprehensive analyses were performed in terms of the phylogenetic tree, structural features, chromosomal localisation, conserved domains, covariance, and cis-acting elements, which can provide a theoretical foundation for further studies on the gene functions and molecular evolutionary mechanisms of the *WRKY* gene family in *P. sibirica*. Furthermore, changes in the expression levels of *PsWRKY* family members in various tissues and under low-temperature stress were investigated, revealing the mechanisms of gene expression regulation in response to low-temperature stress during the growth and development of *P. sibirica*, and providing a theoretical basis for subsequent intensive studies on *WRKYs*.

## Results

### Identification and characterization of *PsWRKY* in *P. sibirica*

*P. sibirica WRKYs* were searched using the Hidden Markov Model (HMM) of the WRKY structural domain (PF03106), and 55 *WRKYs* were identified (Additional file 1: Table S1). Further analysis using Pfam and NCBI-CDD confirmed that all proteins contained the full WRKY domain. We named these genes *PsWRKY1*–*PsWRKY55* according to their respective positions on the chromosomes.

The physicochemical properties of the proteins were analysed, and it was found that the number of amino acids encoded by the *55 PsWRKYs* varied greatly, ranging from 163 (*PsWRKY8*) to 697 (*PsWRKY40*). The relative molecular weights ranged from 18526.84 (*PsWRKY27*) to 75002.92 (*PsWRKY40*) with an average of 42431.12 Da. The theoretical isoelectric point (pI) between 4.78 (*PsWRKY39*) and 10.33 (*PsWRKY5*), and 29 and 26 PsWRKY members were acidic (< 7) and basic (> 7), respectively. The hydrophobicity results were < 0, indicating that all 55 PsWRKY members were hydrophilic proteins with stable performance. Identification of the instability index showed that all proteins except PsWRKY39 (< 40) were unstable, with PsWRKY8 having the strongest hydrophilicity of -1.164. The maximum and minimum aliphatic index values were 79.07 (PsWRKY55) and 42.19 (PsWRKY42), respectively. The predicted subcellular localization revealed that 51 *PsWRKYs* were found in the nucleus; *PsWRKY14*, *PsWRKY16*, and *PsWRKY45* were located in the peroxisomes; and only *PsWRKY39* was located in the chloroplast.

### Multiple sequence alignment and phylogenetic analysis of *PsWRKY* Family

**Fig. 1:**
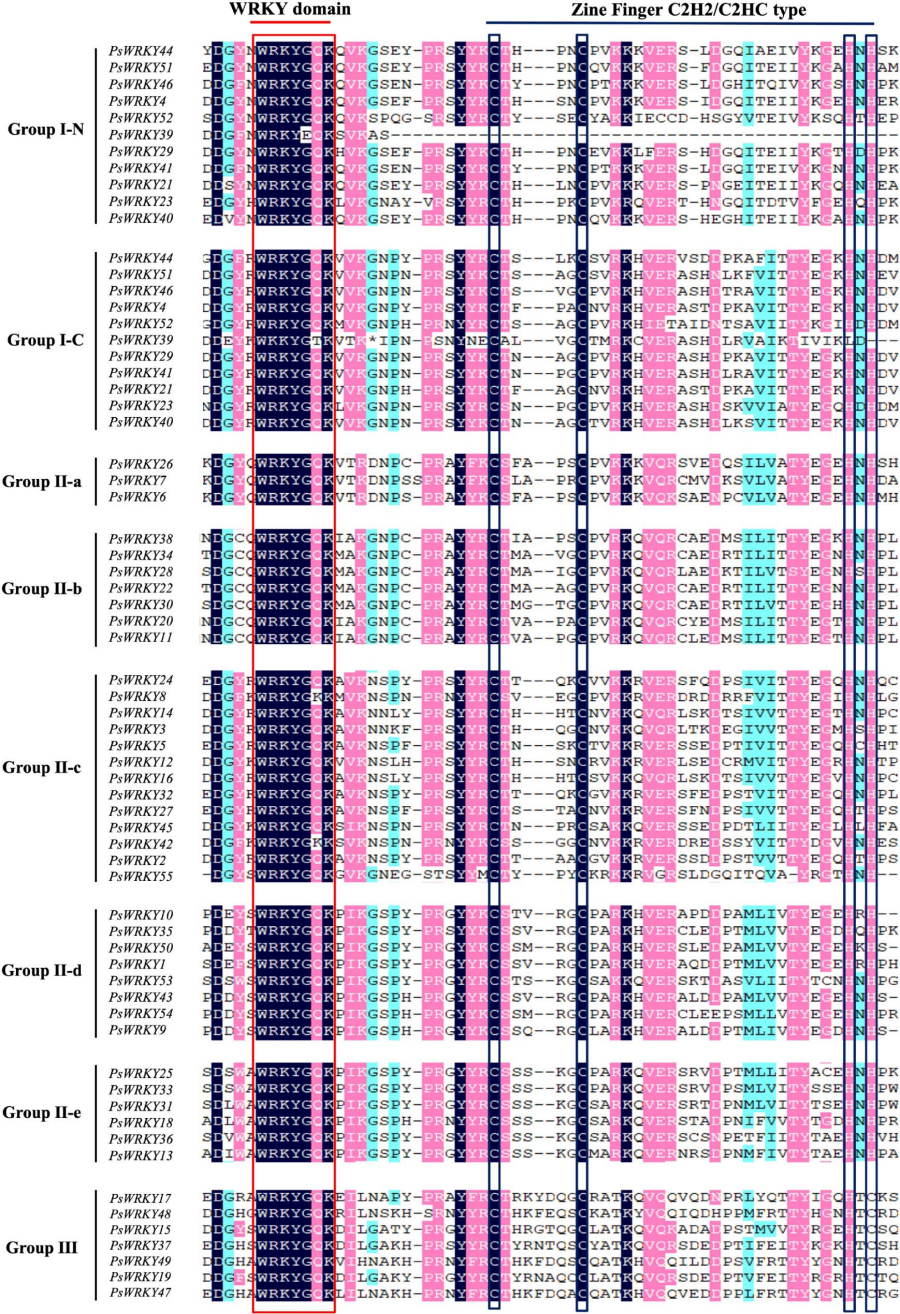
Multiple sequence alignments of conserved domains in PsWRKY transcription factors. Red box represent the conserved WRKY domains, and black box represent the zinc finger motif

The amino acid sequences of the WRKY structural domains of the PsWRKYs were analysed using multiple sequence alignment, and the characteristics of the WRKY structural domains of individual PsWRKYs were determined. The results showed (Fig. 1) that most of the core sequences of WRKY contain complete or nearly complete domains, and individual amino acids of the conserved motifs of PsWRKYs have undergone specific mutations and evolution, among which *PsWRKY8* and *PsWRKY42* underwent single amino acid mutations, and the conserved motif changed from “WRKYGQK” to “WRKYGKK”. Furthermore, two conserved motifs at the N-terminal and C-terminal ends of *PsWRKY39* were changed to “WRKYEQK” and “WKKYGTK”, respectively.

**Fig. 2:**
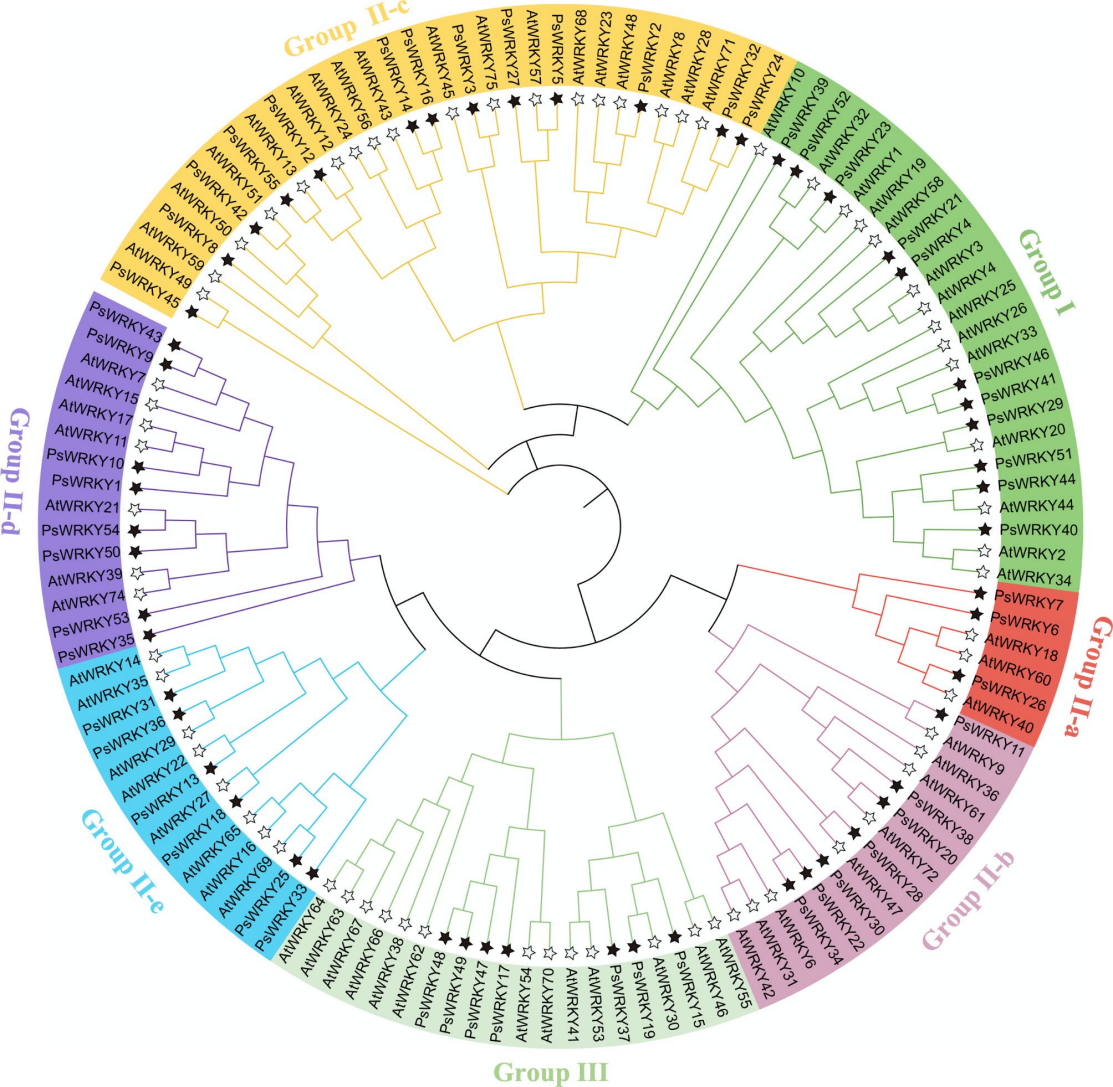
Phylogenetic analysis of *P. sibirica* and *A. thaliana* WRKY domain. Different coloured branches represent different subfamilies. *P. sibirica* and *A. thaliana* are marked as black and white pentagrams respectively

To investigate the affinities and biological connections among members of the *P. sibirica* WRKY family, we selected 71 *AtWRKYs* from *Arabidopsis thaliana* as references. Phylogenetic analysis of *P. sibirica* and *A. thaliana WRKYs* was performed using MEGA 11.0, and the classification of *AtWRKYs* gene families was referred to further classify PsWRKY into three categories: Group I, Group II, and Group III, and Group II was further divided into (II-a, II-b, II-c, II-d, and II-e). Group I had 11 members with two WRKY domains, Group II had 37 members with one WRKY domain, of which Group II-d had one WRKY structural domain in addition to the Pant Zn Cluster transcription factor structural domain. Finally, Group III had seven members with one WRKY domain (Fig. 2). The phylogenetic tree was generated using *WRKYs* from four closely related species (*P. sibirica*, *P. mume*, *Prunus persica*, and *Prunus armeniaca*) to explore the evolution of *WRKY* genes across species (Figure S2). All *WRKYs* from the four species were clustered into the seven classes described above (I, II-a, II-b, II-c, II-d, II-e, III). Also, the phylogenetic tree showed that the number of *WRKYs* in the *WRKY* gene family subgroups was similar in the four species.

### Gene structure and motif composition of *PsWRKYs*

**Fig. 3:**
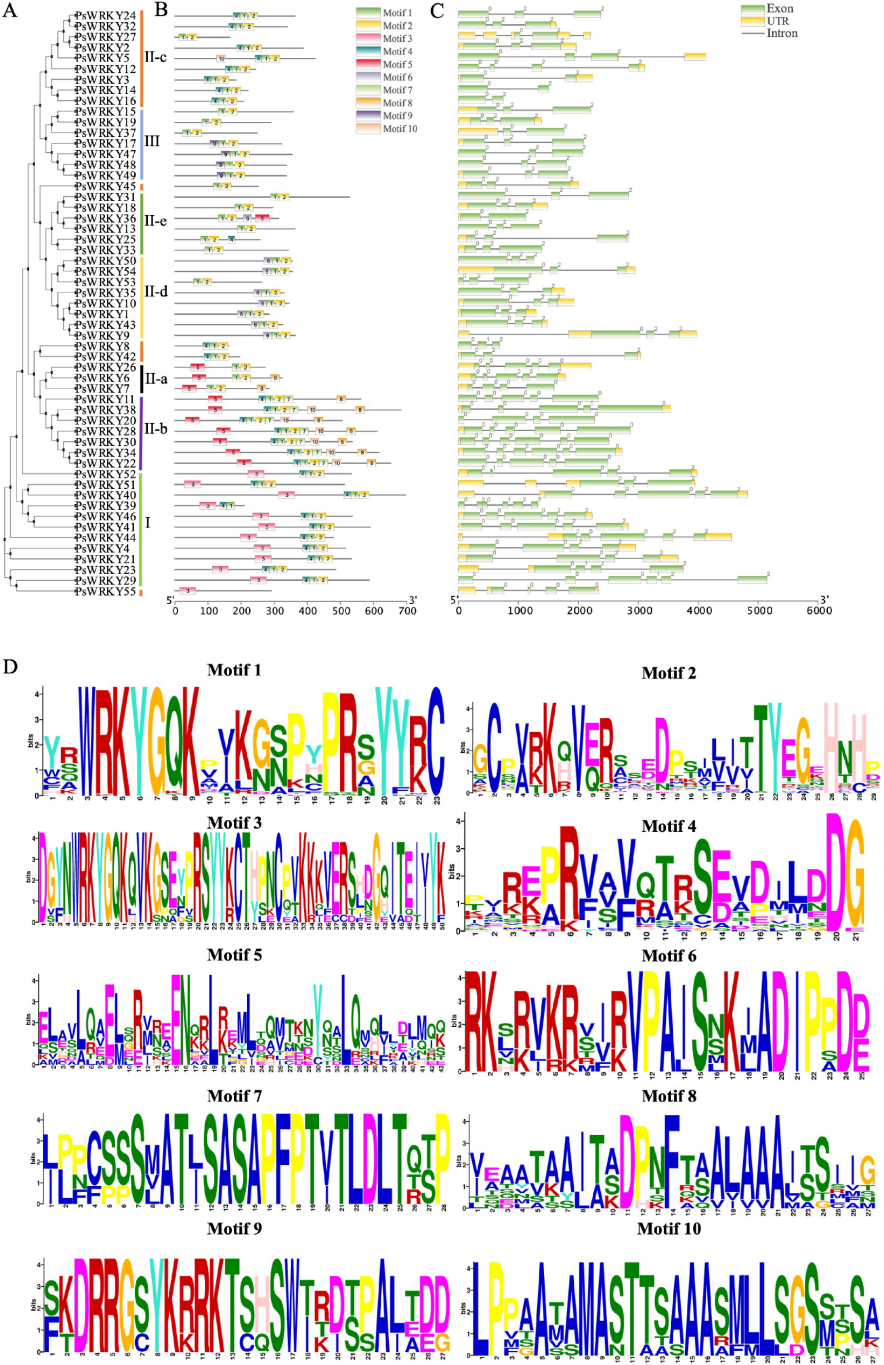
Phylogenetic relationships, conserved motifs, and gene structural analyses of the *PsWRKYs.*

Since the variety of gene structures can indicate the evolution of gene families, we performed a phylogenetic tree construction of 55 *PsWRKY*s (Fig. 3A) and analysed the intron/exon structures and conserved motifs of each *PsWRKY* gene to gain a more in-depth understanding of the *P. sibirica WRKY* family. It was observed that *PsWRKY3*, *PsWRKY14*, *PsWRKY16*, *PsWRKY37*, and *PsWRKY36* contained only two exons, whereas *PsWRKY39, PsWRKY29, PsWRKY30* and *PsWRKY34* contained most exons (Fig. 3C). The number of exons within *PsWRKYs* varied between two and six, with most gene members containing three exons, and 15 *PsWRKY* members have no untranslated region (UTR). The *PsWRKYs* had between one and five introns. Interestingly, except for *PsWRKY36*, the other six members of the group II-e all have three exons and two introns. To identify conserved motifs in all 55 PsWRKYs and gain a better understanding of PsWRKY diversification, we utilized the MEME v5.1.1 program. Our analysis resulted in the annotation of 10 predicted motifs. Figure 3D displays detailed information on the ten motifs. As shown in Fig. 3B, the PsWRKY groups had highly similar conserved motifs. In Group I, 10 of the 11 proteins had structures with motifs 3, 4, 1, and 2 in tandem, whereas one protein (*PsWRKY39*) lacked motif 2. Motif 9 was unique to *PsWRKY17*, *PsWRKY47*, *PsWRKY48*, and *PsWRKY49* in group III, while motif 7 is only distributed in group II-b, and *PsWRKY55* contains only one motif 3.

(A) Neighbour-joining phylogenetic relationships of *PsWRKYs*. (B) Locations of conserved motifs in *PsWRKYs.* The ten predicted motifs are indicated by boxes of various colours. (C) Gene structure of *PsWRKYs*. Black lines and green and yellow boxes indicate introns, exons, and UTR, respectively. (D) Detailed sequence information for motifs 1–10.

### Chromosomal localization, duplication, synteny, and Ka/Ks analysis of *PsWRKYs*

**Fig. 4:**
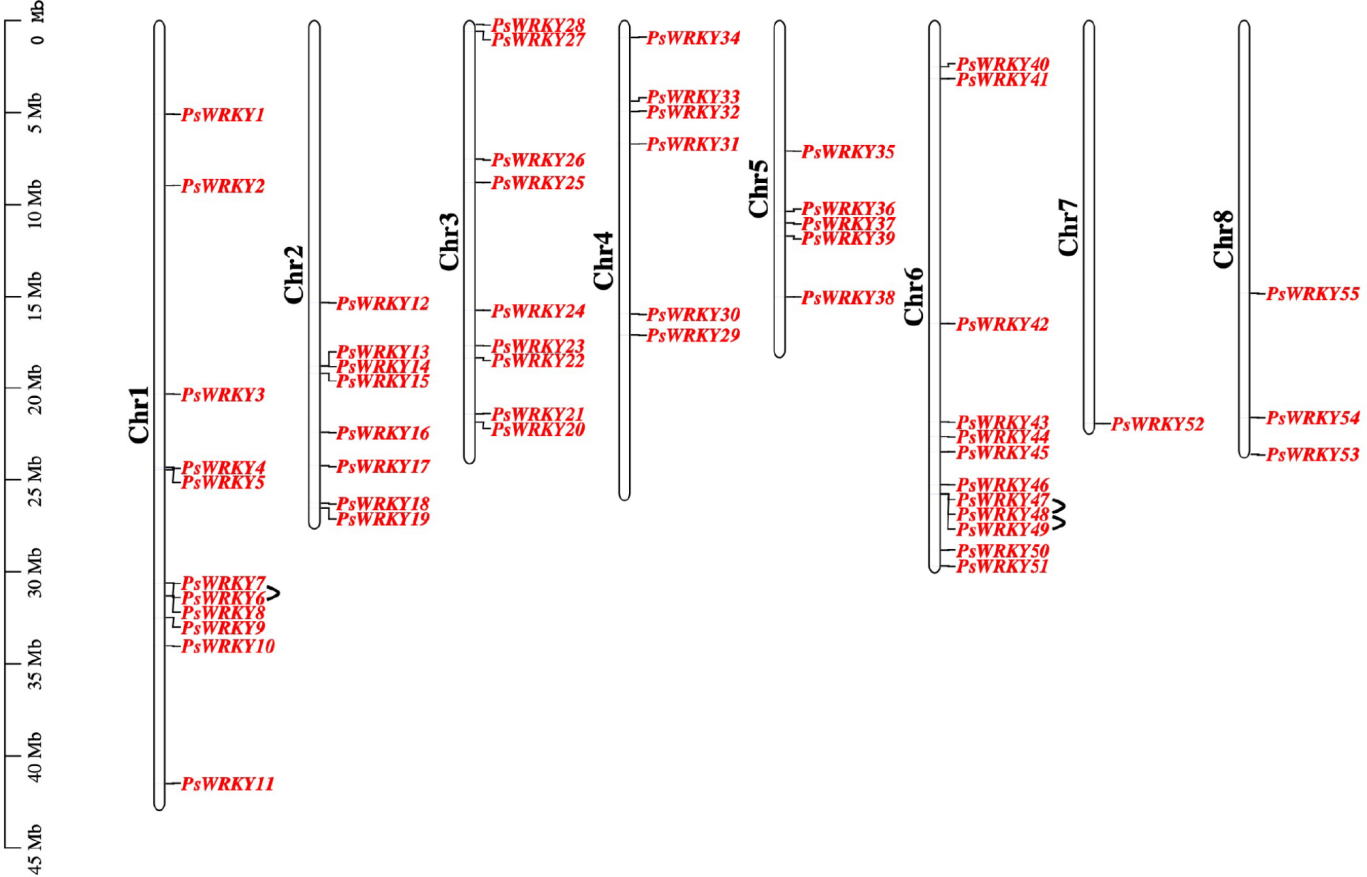
Chromosome distributions of *PsWRKYs*. The black arcs connected were tandem duplication genes

A chromosomal location map was constructed using the genomic information of *P. sibirica* to illustrate the distribution of *PsWRKY* members on chromosomes (Fig. 4). *PsWRKYs* were randomly distributed on eight chromosomes, with most genes concentrated near the caudal middle end of Chr6 (21.8%). Their distribution on Chr1, Chr2, Chr3, Chr4, and Chr5 was 20%, 14.5%, 16.36%, 10. 9%, and 9.1%, respectively. In addition, chromosome 8 contained 5.5% of *PsWRKYs*, and the least *PsWRKYs* were distributed on chromosome 7 (1.8%).

**Fig. 5:**
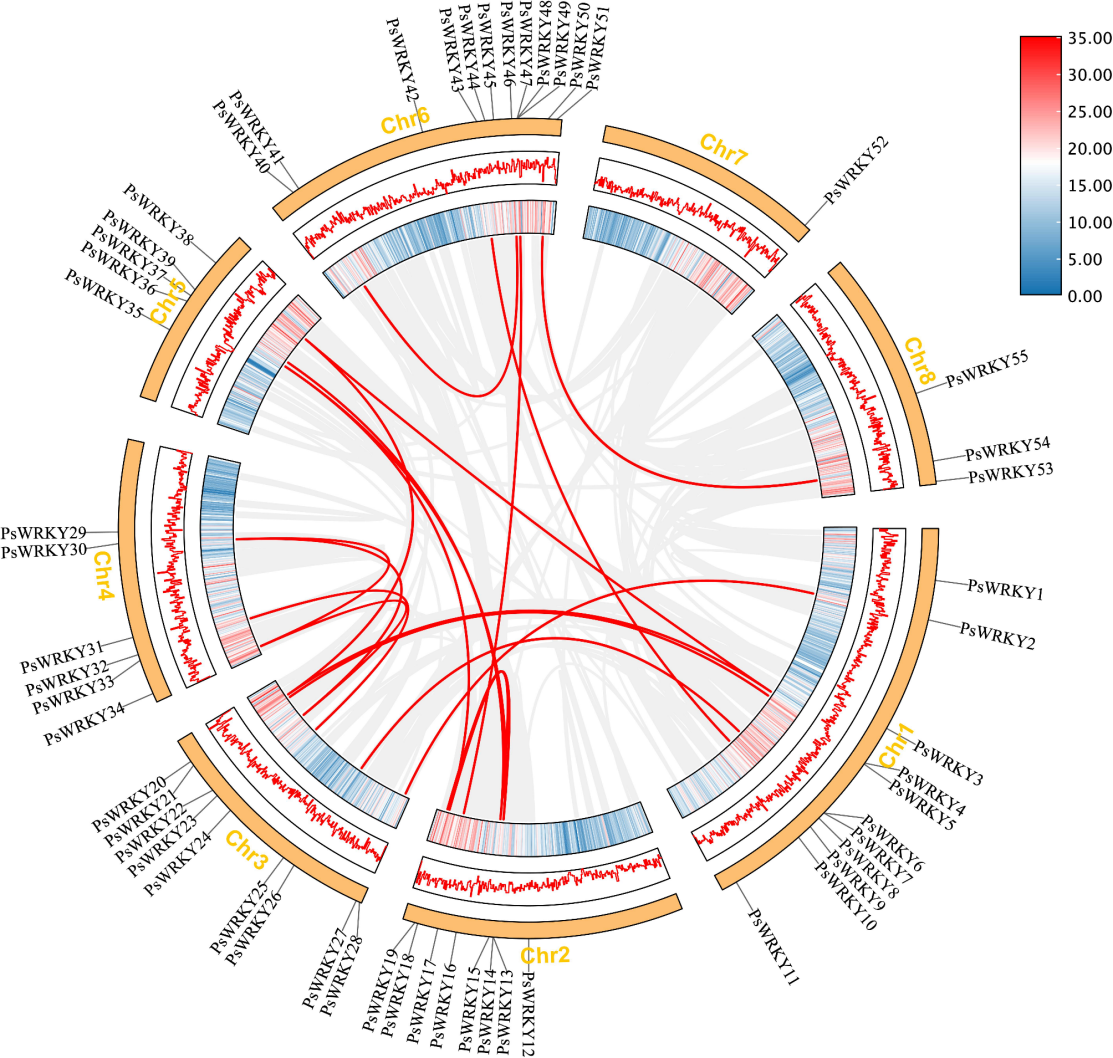
Synteny analysis of *WRKYs* in *P. sibirica* genome

Gene replication patterns (tandem and segmental replication) were analysed using MCScanX. Among the *PsWRKY* gene families, it was found that (*PsWRKY6*, *PsWRKY7*), (*PsWRKY47*, *PsWRKY48*), and (*PsWRKY48*; *PsWRKY49*) were tandem repeat replicates (Fig. 5; Table 1). Additionally, 17 pairs of segmental replication events involving 27 *PsWRKYs* were detected. These findings indicate that these gene clusters were formed due to gene duplication. The *PsWRKY* gene family underwent a few tandem duplication events during evolution, while segmental duplication events played a major role in driving its evolution. In order to assess whether these homologous *PsWRKY* underwent selection pressure, we calculated Ks, Ka, and Ka/Ks. Tandem duplication yielded Ka/Ks ratios of 0.2531–0.3812 and 0.1149–0.3493 for segmental duplication (Fig. 5; Table 1). Except for three co-dominant *PsWRKY* gene pairs, (*PsWRKY9*; *PsWRKY43*), (*PsWRKY1*; *PsWRKY36*), and (*PsWRKY24*; *PsWRKY32*), for which Ka/Ks values were not available, the Ka/Ks ratios of all co-dominant *PsWRKY* gene pairs were less than 1, indicating that these gene pairs were purified and screened.

**Fig. 6:**
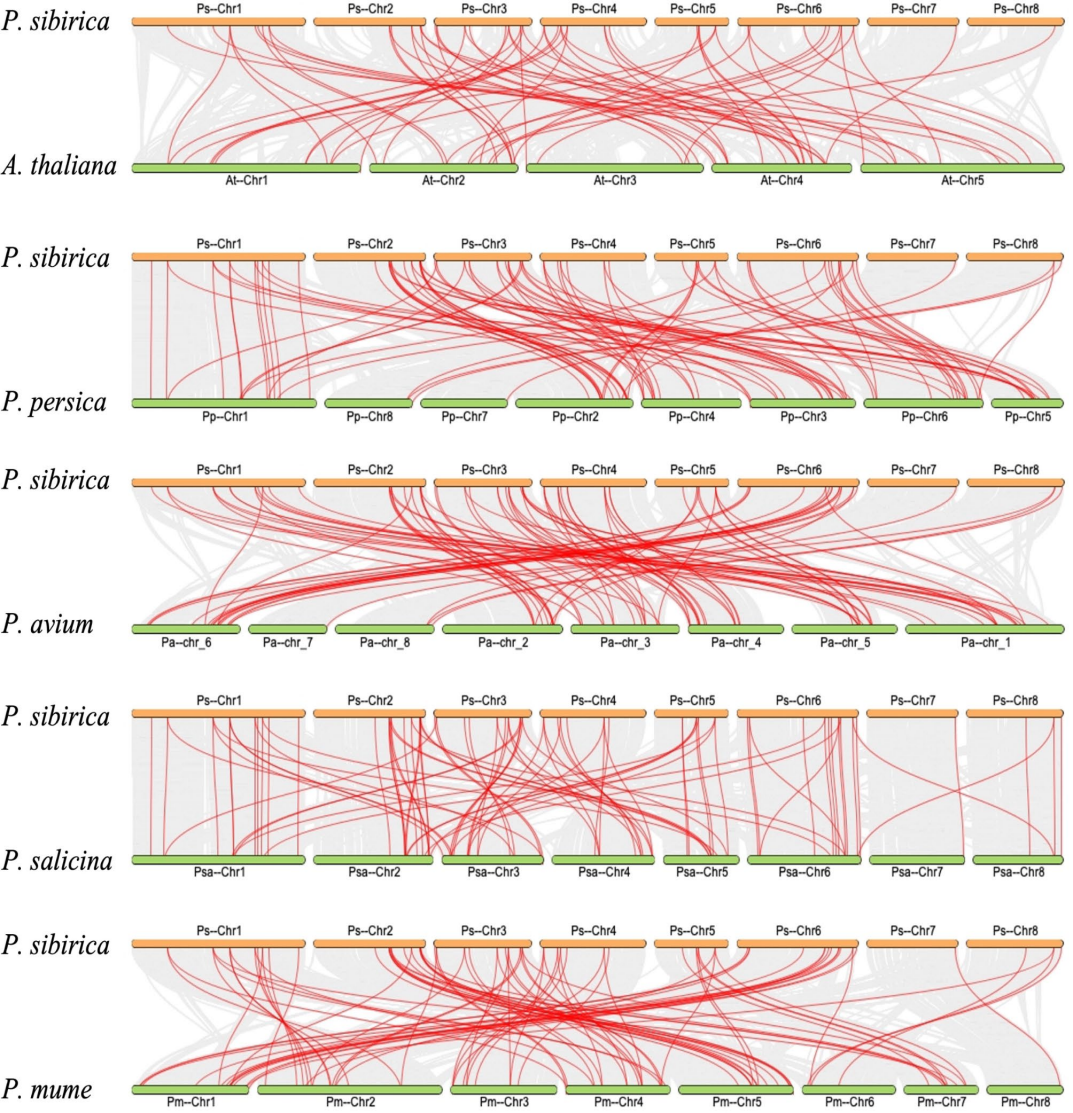
Synteny analysis of *WRKYs* in *P. sibirica* with, *A. thaliana*, *P. persica*, *P. avium, P. salicina*, and *P. mume*. Gray lines represent the collinear blocks within *P. sibirica* and other plant genomes, while red lines emphasize the pairs of syntenic *PsWRKYs*.

To gain further insight into the evolutionary relationships among *WRKY* members in different plant species, a syntenic relationships were traced between *PsWRKYs* and their homologues in other species, including *A. thaliana*, *Prunus salicina*, *P. mume, P. persica*, and *Prunus avium*. The least collinear gene pairs (64 pairs) were between *P. sibirica* and *A. thaliana*, and the most collinear gene pairs (94 pairs) were between *P. sibirica* and *P. salicina*, 86 pairs between *P. mume*, 89 pairs between *P. persica*, and 87 pairs between *P. avium* (Fig. 6).

Gray lines represent all synteny blocks in the *P. sibirica* genome, while red lines indicate segmental duplication genes between *PsWRKYs*. Chromosome gene density is represented by a hot map (inner circle) and column map (middle circle), with the outer circle showing the length of chromosomes.

**Table 1:** Segmental replication and tandem repeat replication covariate gene pair Ka/Ks analysis of *PsWRKYs*

Gene pair name		Gene pair ID		Ka	Ks	Ka/Ks	Duplications type
PsWRKY2	PsWRKY27	PaF106G0100001343.01	PaF106G0300014526.01	0.2657	1.9584	0.1357	segmental duplication
PsWRKY6	PsWRKY26	PaF106G0100004627.01	PaF106G0300013492.01	0.4076	1.2112	0.3365	segmental duplication
PsWRKY4	PsWRKY21	PaF106G0100003357.01	PaF106G0300011494.01	0.2659	1.3083	0.2032	segmental duplication
PsWRKY9	PsWRKY43	PaF106G0100005037.01	PaF106G0600024291.01	0.2851	Na	Na	segmental duplication
PsWRKY15	PsWRKY19	PaF106G0200009172.01	PaF106G0200010641.01	0.5049	2.8421	0.1776	segmental duplication
PsWRKY13	PsWRKY18	PaF106G0200009086.01	PaF106G0200010574.01	0.5506	3.0714	0.1793	segmental duplication
PsWRKY15	PsWRKY37	PaF106G0200009172.01	PaF106G0500019874.01	0.4716	1.5079	0.3128	segmental duplication
PsWRKY13	PsWRKY36	PaF106G0200009086.01	PaF106G0500019756.01	0.4608	Na	Na	segmental duplication
PsWRKY18	PsWRKY36	PaF106G0200010574.01	PaF106G0500019756.01	0.4946	4.3032	0.1149	segmental duplication
PsWRKY17	PsWRKY47	PaF106G0200010134.01	PaF106G0600025042.01	0.5329	2.5901	0.2057	segmental duplication
PsWRKY22	PsWRKY34	PaF106G0300012051.01	PaF106G0400018245.01	0.3184	2.5607	0.1243	segmental duplication
PsWRKY24	PsWRKY32	PaF106G0300012509.01	PaF106G0400017434.01	0.3285	Na	Na	segmental duplication
PsWRKY22	PsWRKY30	PaF106G0300012051.01	PaF106G0400015665.01	0.3131	1.9535	0.1603	segmental duplication
PsWRKY20	PsWRKY38	PaF106G0300011395.01	PaF106G0500020691.01	0.4278	1.2246	0.3493	segmental duplication
PsWRKY34	PsWRKY30	PaF106G0400018245.01	PaF106G0400015665.01	0.4189	2.0275	0.2066	segmental duplication
PsWRKY41	PsWRKY46	PaF106G0600021917.01	PaF106G0600024936.01	0.2939	1.4943	0.1967	segmental duplication
PsWRKY50	PsWRKY54	PaF106G0600025642.01	PaF106G0800029773.01	0.2719	1.3541	0.2010	segmental duplication
PsWRKY6	PsWRKY7	PaF106G0100004627.01	PaF106G0100004628.01	0.6564	2.5930	0.2531	tandem duplication
PsWRKY47	PsWRKY48	PaF106G0600025042.01	PaF106G0600025043.01	0.2629	0.4680	0.5618	tandem duplication
PsWRKY48	PsWRKY49	PaF106G0600025043.01	PaF106G0600025044.01	0.1044	0.2739	0.3812	tandem duplication

Note: Ka, non-synonymous substitution rate; Ks, synonymous substitution rate; Ka/Ks, selection pressure ratio; Na, no result

### Analysis of *PsWRKY cis*-acting elements

**Fig. 7:**
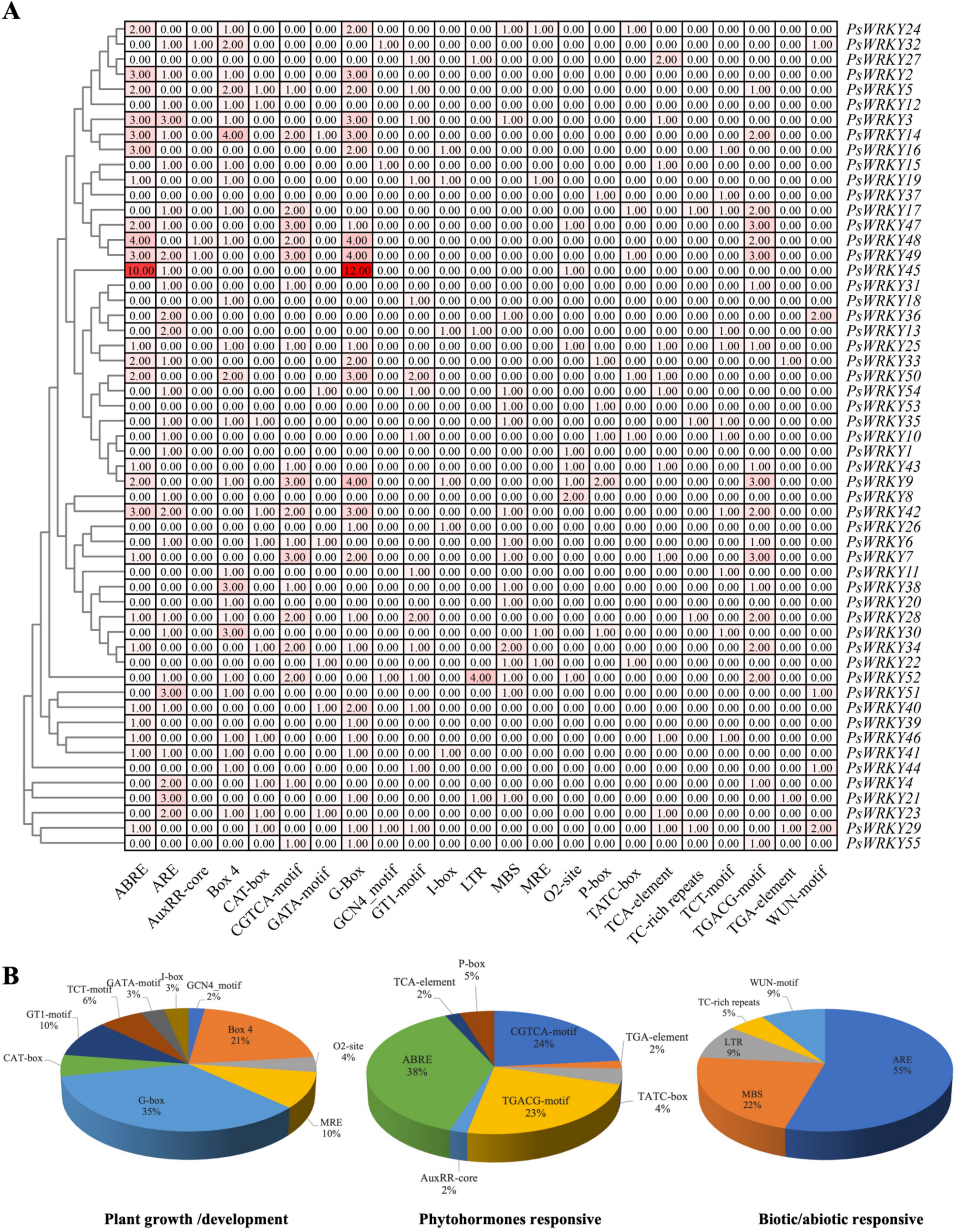
Analysis of the cis-acting elements of the *PsWRKYs*.

In order to determine the specific types and distribution of cis-acting elements present in the promoters of *PsWRKYs*, an analysis of promoter cis-acting elements was conducted using PlantCARE. From the predicted results, 23 representative cis-acting elements were selected for functional analysis (Fig. 7 and S1). Cis-acting elements are classified into three major categories according to their regulatory functions: plant growth/development, phytohormone responses, and biotic/abiotic stress responses. The first category of cis-acting elements for plant growth and development prevalent in the promoter region included the GCN4_motif, MRE, Box-4, CAT-box, O2-site, G-box, GT1-motif, TCT-motif, GATA-motif, and I-box (Fig. 7A, B). Among these, the GCN4_motif, O2-site, and CAT box are involved in endosperm expression, metabolic regulation of maize alcohol-soluble proteins, and meristematic tissue expression, respectively. In addition, the promoter regions of almost all *PsWRKYs* contained at least one light-response element, with the highest percentage in G-box (35%), followed by Box-4, GT1-motif, TCT-motif, GATA-motif, MRE, and I-box (Fig. 7B). Among the second class of phytohormone response elements, TGA-element (2%) and AuxRR-core (2%), were involved in growth hormone response, TATC-box (4%) and P-box (5%) in gibberellin response, ABRE (38%) in abscisic acid response, TCA-element (2%) in salicylic acid response, and CGTCA- motif (24%) and TGACG-motif (23%) in methyl jasmonate (MeJA) responsive (Fig. 7B). The third category includes biotic/abiotic stress response elements like LTR, ARE, MBS, WUN-motif, and TC-rich repeats, which are associated with cold stress, anaerobic induction, drought stress, mechanical damage, defense, and stress responses, respectively. In *P. sibirica*, ARE was present in 51 *PsWRKY*s, the Wun motif was present in four *PsWRKYs*, and LTR elements were found in four promoters of *PsWRKY*s. *PsWRKY52* was strongly induced by cold stress and contained four LTR elements. Taken together, *PsWRKYs* may be associated with light, hormone, stress responses, and growth and developmental pathways in *P. sibirica*.

(A) Different colours represent the number of cis-acting elements. (B) The Pie chart size represents the percentage of promoter elements in each category, such as phytohormone-responsive, biotic/abiotic stress, and plant growth and development.

### Gene ontology annotation analysis of *PsWRKYs*

**Fig. 8:**
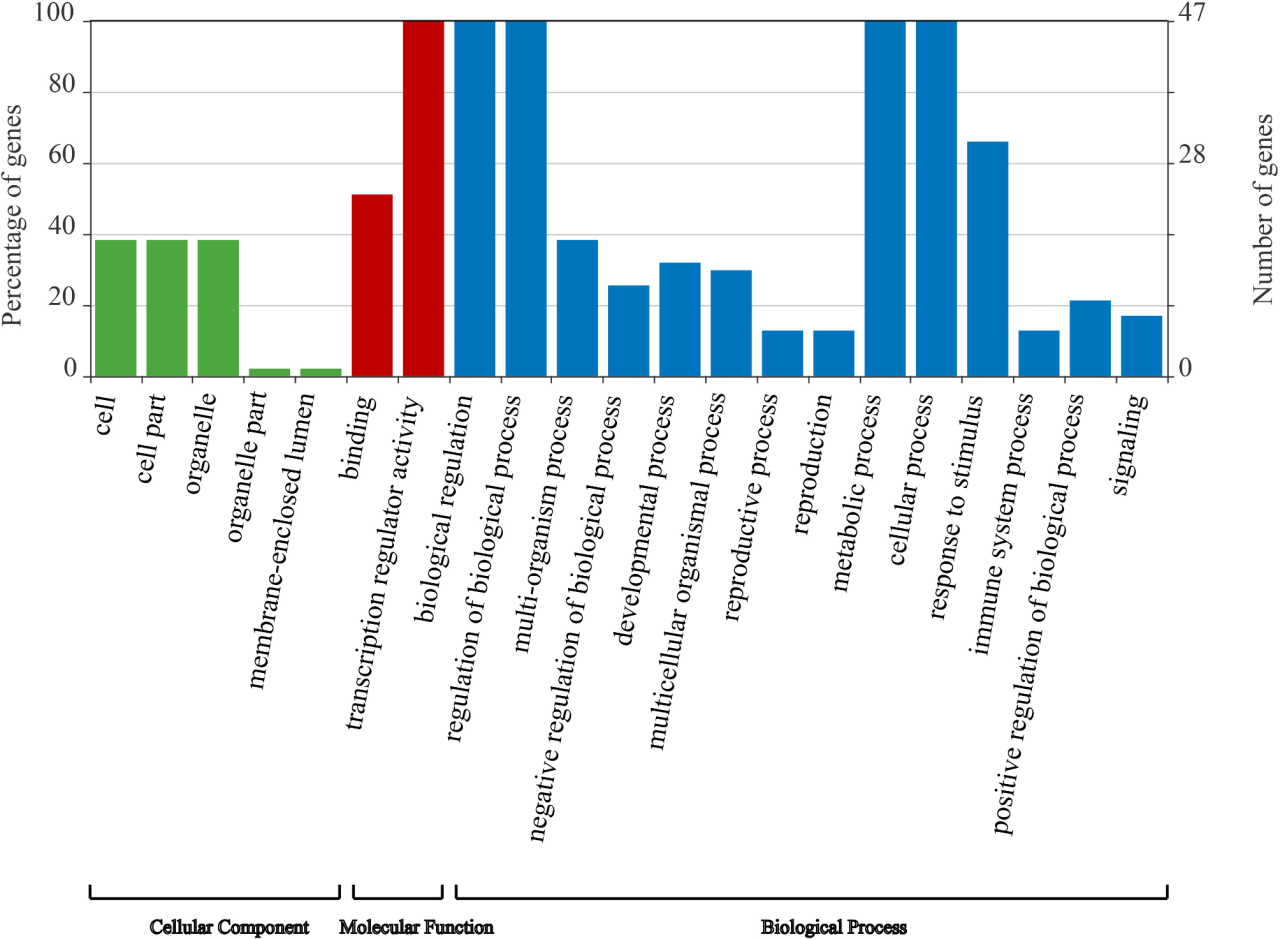
Gene ontology (GO) analysis of *PsWRKY*s.

To better inform the transcription factor functions of PsWRKYs, the EggNog website was used for gene ontology (GO) annotation analysis. The 47 PsWRKYs were classified into 21 functional groups according to their protein sequence similarity and into three classes: biological processes, molecular functions, and cellular components (Fig. 8). Within the category of biological processes, the majority of *PsWRKYs* were engaged in the regulation of cellular processes (GO:0009987), biological processes (GO:0050789), biological (GO:0065007), and metabolic (GO:0008152) processes. Furthermore, six (*PsWRKY2*, *PsWRKY24*, *PsWRKY32*, *PsWRKY47*, *PsWRKY48*, and *PsWRKY49*) (GO:0032502), 11 (*PsWRKY3, PsWRKY4, PsWRKY6, PsWRKY7, PsWRKY17, PsWRKY19, PsWRKY26, PsWRKY32, PsWRKY47, PsWRKY48*, and *PsWRKY49*) (GO:0050896), six (*PsWRKY2, PsWRKY19, PsWRKY32, PsWRKY47, PsWRKY48*, and *PsWRKY49*) (GO:0002376), and six *PsWRKYs* (*PsWRKY31, PsWRKY32, PsWRKY40, PsWRKY44, PsWRKY51*, and *PsWRKY53*) (GO:0022414) were projected to be engaged in the developmental, response to stimuli, immune system response, and reproductive processes, including fruit and seed development, respectively. The molecular functions of *PsWRKYs* were associated mostly with ‘binding’ (GO:0005488) and ‘transcription regulator activity’ (GO:0140110). The cellular components of *PsWRKYs* include organelles (GO:0043226), cell parts (GO:0044464), and cells (GO:0005623).

### Analysis of *PsWRKYs* expression in different tissues

**Fig. 9:**
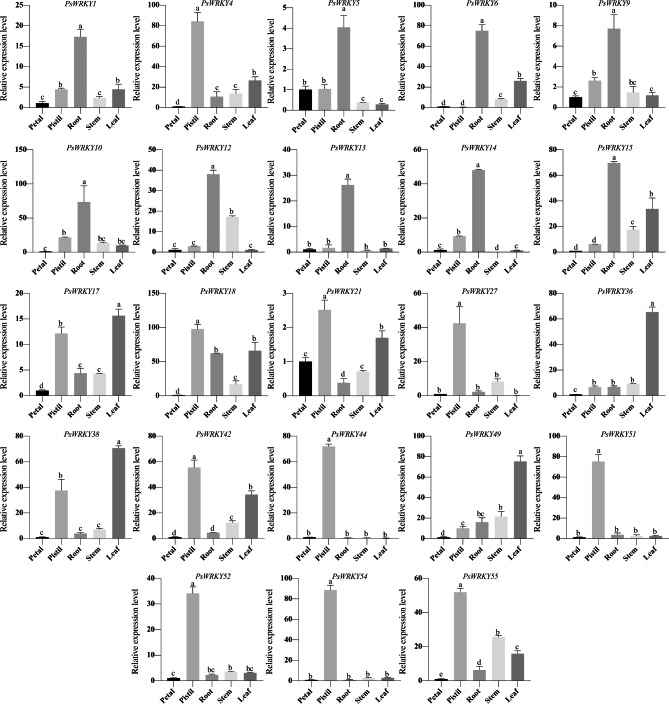
Expression analysis of the 23 *PsWRKY*s using quantitative real-time PCR (qRT-PCR) in different tissues of *P. sibirica*

The integrity and quality of the extracted total RNA was assessed using Agilent 2100 bioanalyzer (Agilent Technologies), all RNA samples had RNA integrity scores greater than 7 (Table S4), indicating that the extracted total RNA was of high quality and could be subjected to subsequent quantitative real-time PCR. To characterise the potential functions of *PsWRKYs*, we screened 23 *PsWRKYs* that could potentially be associated with plant growth development using promoter cis-acting elements, GO annotation analysis, and *WRKY* homeotic genes in other plants (Table S3) and examined their expression levels in different tissues of *P. sibirica* using qRT-PCR (Fig. 9). The 23 *PsWRKYs* showed different expression patterns in various tissues. Firstly, ten genes were highly expressed in the pistil, including *PsWRKY4*, *PsWRKY18*, *PsWRKY21*, *PsWRKY27*, *PsWRKY42*, *PsWRKY44*, *PsWRKY51*, *PsWRKY52*, *PsWRKY54*, and *PsWRKY55*. Among them, the expression of *PsWRKY4*, *PsWRKY44*, *PsWRKY51*, *PsWRKY52*, and *PsWRKY54* in the pistil was much higher than that in other tissues. Nine *PsWRKYs* (*WRKY1*, *WRKY5*, *WRKY6*, *WRKY9*, *WRKY10*, *WRKY12*, *WRKY13*, *WRKY14*, and *WRKY15*) were dominantly expressed in the roots. Notably, *PsWRKY6*, *PsWRKY10*, and *PsWRKY15* were 75-, 70-, and 70-fold more highly expressed, respectively, in roots than in petals. Third, in the leaf, the dominantly expressed *PsWRKYs* were *PsWRKY17*, *PsWRKY36*, *PsWRKY38*, and *PsWRKY49*.

Total RNA was extracted from the petal, pistil, root, stem, and leaf. Petal was used as an internal control and normalized to 18 S rRNA levels. The standard error was represented by error bars and the significant differences were identified using ANOVA followed by Duncan’s multiple range test (p < 0.05, n = 3), where different letters indicate significant differences.

### Analysis of *PsWRKYs* expression under low-temperature stress

**Fig. 10:**
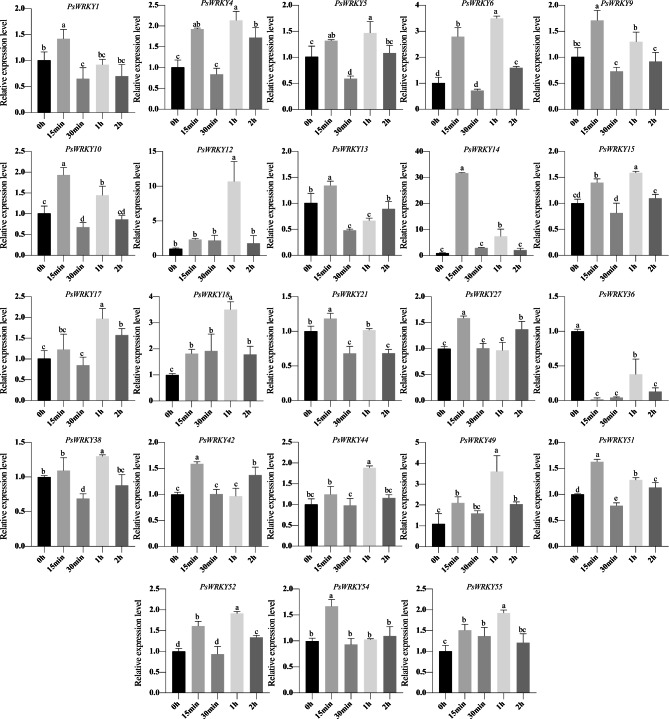
Expression analysis of the 23 *PsWRKY*s in *P. sibirica* pistils under low-temperature stress

*WRKY* transcription factors play an important regulatory role in the response of plants to low-temperature stress. To investigate the expression pattern of *PsWRKYs* that may be associated with the low-temperature response, qRT-PCR was used to observe the expression levels of 23 *PsWRKYs* at different stages (0 h, 15 min, 30 min, 1 h, and 2 h) of low-temperature stress (-4 °C). The results showed that 23 *PsWRKYs* showed significant differences, with most of them showing high expression after 15 min and 1 h of low-temperature stress (Fig. 10). Among them, 10 genes were highly expressed after 15 min of low-temperature stress, including *PsWRKY1*, *PsWRKY9*, *PsWRKY10*, *PsWRKY13*, *PsWRKY14*, *PsWRKY21*, *PsWRKY27*, *PsWRKY42*, *PsWRKY51*, and *PsWRKY54*; 12 *PsWRKYs* (*PsWRKY4*, *PsWRKY5*, *PsWRKY6*, *PsWRKY12*, *PsWRKY15*, *PsWRKY17*, *PsWRKY18*, *PsWRKY38*, *PsWRKY44*, *PsWRKY49*, *PsWRKY52*, and *PsWRKY55*) were expressed highest after 1 h of low-temperature stress. Notably, the largest increase in expression was detected for *PsWRKY14* (approximately 30-fold) after 15 min of low-temperature stress, whereas *PsWRKY12* expression was upregulated 10-fold after 1 h of low-temperature stress.

The relative expression levels of *23 PsWRKYs* were examined by qRT-PCR at -4 °C treatment for 0 h, 15 min, 30 min, 1 h, and 2 h. The 0 h was used as a control and 18 S rRNA as an internal reference gene.The standard error was represented by error bars and the significant differences were identified using ANOVA followed by Duncan’s multiple range test (p < 0.05, n = 3), where different letters indicate significant differences.

## Discussion

*WRKYs* are one of the largest transcription regulatory factor families in plants, and play a crucial role in plant growth, development, and defense mechanisms [4]. In the present study, 55 *WRKYs* were identified throughout the genome of *P. sibirica* using bioinformatic analysis. The number of *PsWRKYs* was similar to the identified *WRKYs* in *P. mume* (58) [37], *P. persica* (58) [29], and *P. armeniaca* (56) [30]; however, it was significantly less than that of model plants, like Arabidopsis (72) [31] and tomato (88) [32], and the difference in number was most pronounced in Groups II-c and III, suggesting that *WRKY* members in these groups had more severe gene loss during evolution, similar to that observed in *P. mume* [37] and *P. armeniaca* [30] *WRKY* gene family.

In this study, analysis of the physicochemical properties of PsWRKYs revealed that the number of amino acids and relative molecular weight of the 55 PsWRKYs varied greatly and that the *PsWRKY* gene family were all hydrophilic proteins, with 52.72% of the acidic proteins and 47.27% of the basic proteins. This indicates that there are differences in the physicochemical properties of *PsWRKY* gene family members. PsWRKY40 contains the largest number of amino acids and may form a complex protein structure and function, but more in-depth analysis and study of its amino acid sequence and combination is needed to fully understand the properties of this protein. Among them, PsWRKY39 is the only stable protein, which may be responsible for biological functions such as maintenance of tissue structure and metabolic regulation. The predicted subcellular localisation of the 55 *PsWRKYs* indicated that most *WRKYs* (51) were located in the nucleus, indicating that their functions may be closely linked to the regulation of target gene expression. Notably, *PsWRKY14, PsWRKY16, and PsWRKY45* are localised in the peroxisome, and the peroxisome may play a crucial role in the cold stress response [33], so the role of these genes in cold stress in *P. sibirica* is of interest to us. Furthermore, *PsWRKY39* is localised in the chloroplast and is speculated to be involved in chloroplast formation, thereby affecting photosynthesis. Phylogenetic analysis based on *AtWRKYs* divided the 55 *PsWRKYs* into three groups, with the largest proportion of *PsWRKYs* in Group II (67%), suggesting that this taxon may have undergone gene duplication events during evolution in *Hypericum perforatum* [34], *Siraitia siamensis* [35] and *P. armeniaca* [30]. Group II was subdivided into five subgroups in which members II-e and II-d were concentrated in two adjacent branches of the same subgroup, as were members of II-a and II-b. This indicates a closer evolutionary relationship between them, consistent with the evolutionary findings of other studies on eggplant [36] and plum [37].

The *WRKY* transcription factor is named after its highly conserved sequence (WRKYGQK) which is crucial for its ability to recognize and bind to the W-box element in target gene promoters [3, 38]. Previous studies have identified variants of the conserved WRKYGQK motif in multiple species. Two variants (WKKYGKK and WKKYGEK) have been reported for *Taraxacum kok-saghyz* [39], while *Ginkgo biloba* has four variants: WRKYAQK, WRKYGEK, WRKYGRK, and WRKYGKK [40]. Moreover, three variants have been observed in *Kandelia obovata* (WRKYGEK, WRKYGKK, and WRKYGQK) [41] and one in *Cannabis sativa* (WRKYGKK) [42]. Variants of WRKYGKK were noted in *PsWRKY8* and *PsWRKY42* protein sequences of Group II-c. Variations in this conserved structural domain may affect the binding specificity of *WRKYs* to downstream target genes, resulting in altered gene functions [43]. For example, the *NtWRKY12* protein with a WRKYGKK motif in *Nicotiana tabacum* can bind to WK-box elements but not to W-box motifs. Further verification is required to determine whether *PsWRKY8* and *PsWRKY42* bind to the W-box [44]. Notably, variations in WKKYGTK and WRKYEQK were observed in the *PsWRKY39* protein sequence of Group I. WKKY variation is common in Asteraceae and has been observed in different legumes, but at a lower frequency. However, no WKKY taxa have been reported in other plant species [45]. Therefore, the functional and DNA-binding properties of *PsWRKY39* require further analysis.

Upon analysing the conserved structural domains showed that the number and type of conserved motifs differed between *PsWRKYs*. However, gene members of the same subgroup exhibited similar conserved motif types, indicating the presence of analogous structures and functions within the same WRKY group [46]. All *PsWRKYs* contained motifs 1 and 2, which could be key elements retained in evolution. Motif 9 is exclusive to Group III, while motifs 6, 7, and 10 are only found in Group II-b. The similarity between conserved motifs in II-a and II-b suggests a close evolutionary relationship. A similar motif composition within the same group or subgroup indicates that the proteins are structurally conserved and may have similar functions [47].

Gene duplication events are the primary drivers for acquiring new genes and enabling the expansion of an organism’s gene family, which consists of two main forms: tandem and segmental duplication [48]. In this study, we found that among the 55 *PsWRKYs*, only three pairs of genes, (*PsWRKY6*; *PsWRKY7*), (*PsWRKY47*; *PsWRKY48*), and (*PsWRKY48*; *PsWRKY49*), showed tandem duplications. A total of 17 *PsWRKYs* had segmental duplications between them, which far outnumbered tandem duplications; these gene pairs were distributed on distinct chromosomes, indicating that segmental duplication at these positions may contribute to the extension of the *PsWRKY* family. Ka/Ks analysis of the identified homologous gene pairs showed that all *PsWRKY* gene family members underwent purifying selection, indicating that the *PsWRKY* gene family evolved to be highly conserved. Based on this, we constructed a collinearity map of the *PsWRKY* family in *A. thaliana*, *P. salicina*, *P. mume*, *P. persica*, and *P. avium. P. sibirica* and *A. thaliana*, *and P. salicina*, *P. mume*, *P. persica*, and *P. avium* there were 64, 92, 86, 89, and 87 collinear gene pairs, respectively. These results indicate that *P. sibirica* shares more collinear connections with other Rosaceae genomes compared to *A. thaliana*, implying a possible evolutionary relationship between them. The large number of collinear pairs shared between *P. sibirica* and *P. salicina* suggests that these two species have a comparatively close evolutionary relationship.

Transcription factors perform crucial functions in signal transduction and the induction of downstream functional gene expression by specifically binding to gene promoter regions and activating or repressing the transcription of downstream genes [49]. The promoter regions of *PsWRKYs* were found to contain numerous cis-elements with multiple potential regulatory functions. Among these cis-elements, some were overrepresented in the *PsWRKYs*, including G-box and Box 4, which regulate plant growth and development, and biotic/abiotic response-associated elements, such as ARE, MBS, WUN-motif, and LTR, followed by involvement in phytohormone response elements, among which signalling molecule elements, such as ABRE, TGACG-motif, and CGTCA-motif, were the most prominent, sensing and transmitting signals in response to external environmental changes, and maintaining plant homeostasis through hormonal networks [50]. These results suggest the functional diversity of *PsWRKYs*. An LTR cis-acting element was found in four *WRKYs*, among which *PsWRKY52* contained four LTR cis-acting elements, which simultaneously verified the adaptation of the gene to a freezing point environment.

Gene expression is closely related to gene function [51]. Research has demonstrated that the expression of *WRKYs* in specific tissues has a significant impact on the growth and development of plants by controlling the expression of genes engaged in growth and differentiation [52]. In this study, many *PsWRKYs* were constitutively expressed in the pistils and roots of *P. sibirica*, and numerous of these, for instance, *PsWRKY6*, *PsWRKY13*, *PsWRKY14*, *PsWRKY27*, *PsWRKY44*, *PsWRKY51*, *PsWRKY52*, and *PsWRKY54*, showing tissue-specific expression patterns, meaning that *PsWRKYs* play crucial roles in various aspects of plant development and exhibit distinct functions in different tissue types. *P. sibirica* is an early spring flowering species, and frost damage to its floral organs is a significant constraint to almond production. Among apricot floral organs, the pistil is the least resistant to frost and directly affects fruit yield [53]. In our study, *PsWRKYs* were predominantly expressed in the pistils, with 10 being highly regulated. *PsWRKY4, PsWRKY18, PsWRKY21, PsWRKY27, PsWRKY42, PsWRKY44, PsWRKY51, PsWRKY52, PsWRKY54*, and *PsWRKY55* were the predominant transcripts that accumulated in pistils, indicating that these *PsWRKYs* are essential in pistil growth and development. The results of GO annotation analysis confirmed the possible involvement of *PsWRKY44* and *PsWRKY51* in pistil development and fruit formation in *P. sibirica*. This was consistent with the results of previous studies on *Juglans regia* [54]. The root system plays a crucial role in the uptake of water and nutrients in plants. Consequently, it is often the first to react to stressful conditions such as drought, high salt levels, and other external factors. In this study, nine *PsWRKY* genes, including *PsWRKY1, PsWRKY5, PsWRKY6, PsWRKY9, PsWRKY10, PsWRKY12, PsWRKY13, PsWRKY14*, and *PsWRKY15*, were highly expressed in roots, suggesting that *P. sibirica WRKY* may play a specific role in root growth and development. Studies have suggested that *AtWRKY75* is implicated in regulating the nutrient starvation response and root development [55], and *AtWRKY46* inhibits ammonium efflux from the root elongation zone to promote ammonium tolerance in Arabidopsis [56]. The results of the phylogenetic analysis indicated that *PsWRKY14* exhibited a closer genetic relationship with *AtWRKY75*, while *PsWRKY15* was found to be more closely related to *AtWRKY46*, indicating that these *PsWRKYs* may be involved in regulating root growth and material uptake in *P. sibirica*; however, the related mechanisms need to be further studied.

The *WRKY* family has been linked to both biotic and abiotic stress responses [38]. Research has demonstrated the significant regulatory role of the *WRKY* family in plant responses to low-temperature stress. For example, in rice, *WRKY53* negatively affects cold tolerance during gestation by repressing GA transcription in anthers [57], and overexpression of *KoWRKY40* and *VvWRKY28* in Arabidopsis enhances cold tolerance in transgenic Arabidopsis [58, 59]. In our study, we examined the expression patterns of *PsWRKYs* in *P. sibirica* pistils under low-temperature stress. Our findings revealed that out of the 23 *PsWRKYs* analysed, most genes were upregulated in response to the stress, while only *PsWRKY36* was downregulated. Similar results have been found in *K. obovata* [41], *Coffea canephora* [47], and *P. mume* [37], suggesting that *PsWRKY* transcription factors may have positive or negative regulatory effects. Significantly, seven *PsWRKYs* (*PsWRKY4*, *PsWRKY6*, *PsWRKY12*, *PsWRKY14*, *PsWRKY18*, *PsWRKY49*, and *PsWRKY52*) were upregulated more than 2-fold after low-temperature stress, suggesting that these *PsWRKYs* are likely to have a positive function in the low-temperature stress response. *PsWRKY14* was upregulated by approximately 30-fold after 15 min of low-temperature stress, and its relative phenotype was higher in pistils, indicating that it might have a significant effect on pistil freezing resistance. Notably, the cis-acting element prediction results of *PsWRKY52* contained more LTR cis-acting elements, while GO annotation results showed that *PsWRKY4*, *PsWRKY6*, and *PsWRKY49* were engaged in the regulation of the stimulus response, demonstrating that these genes might have major roles in the freezing resistance of *P. sibirica* organs and could be used in future gene function validation. In addition, 10 *PsWRKYs* were highly expressed after 15 min of low-temperature treatment, and 12 *PsWRKYs* were highly expressed after 1 h of low-temperature stress, suggesting that these *PsWRKY* transcription factors may play different roles at different times during the stress response. Recent studies have shown that overexpression of *PmWRKY57* augments tolerance to cold stress in Arabidopsis [16], and sequence comparison has revealed that *PsWRKY5* and *PmWRKY57* are homologous genes, implying that *PsWRKY5* may have an essential function in the response to cold stress and may be associated with cold resistance in *P. sibirica*.

## Conclusions

In summary, 55 *WRKYs* were identified in the *P. sibirica* genome, all of which are located on eight chromosomes. By sequence alignment and phylogenetic analysis, *PsWRKYs* were classified into seven subgroups, and these subgroups shared similarities in their gene structures. Segmental duplication significantly contributed to the expansion of the *P. sibirica WRKY* gene family. Evolutionary difference analysis (Ka/Ks) indicated that *PsWRKYs* were strongly subjected to purifying selection during evolution. GO annotation results indicated that *WRKYs* were primarily involved in the regulation of molecular functions and biological processes, such as transcriptional regulatory activity, response to stimuli, immune system processes, reproductive processes, regulation of metabolic processes, and regulation of cellular processes. The qRT-PCR analysis revealed that 23 *PsWRKYs* were highly expressed in one or more tissues. The *PsWRKYs* were significantly highly expressed after 15 min and 1 h of low-temperature stress. The results presented in this study serve as a foundation for understanding the function of the *WRKY* gene in enhancing plant cold tolerance and the associated molecular mechanisms.

## Materials and methods

### Plant material and treatment

*P. sibirica* clone No. 453 (Appraisal of improved varieties of forest tree, S-SV-PS-002-2021, Liaoning Provincial Department of Forest and Grassland) from the National Forest Germplasm Resource Preservation Repository for *Prunus* species at Shenyang Agricultural University (Kazuo, Liaoning, China) was used as the experimental material. The leaf, stem, root, petal, and pistil tissues of *P. sibirica* clone No. 453 were sampled in the absence of mechanical damage or pests. Additionally, whole plants of *P. sibirica* clone No. 453 were placed in an artificial frost chamber at -4 °C, and pistils were taken as samples after 0 h, 15 min, 30 min, 1 h, and 2 h of stress, with 0 h as control, placed in liquid nitrogen, and stored at -80 °C. The sampling and stress treatment experiments were performed in triplicate.

### Identification and characterization of *PsWRKYs*

*P.sibirica* genome data were downloaded from the Rosaceae genome database (https://www.rosaceae.org/Analysis/10254124), and *A. thaliana* WRKY protein sequences were downloaded from The Arabidopsis Information Resource (TAIR) database (https://www.arabidopsis.org/). A Hidden Markov Model file (PF03106) for *WRKY* was downloaded from the Pfam database (https://www.ebi.ac.uk/interpro/entry/pfam/PF03106/). HMMER 3.0 (http://hmmer.janelia.org/) was used to screen for candidate *PsWRKYs* [60]. Two online software programs, the Pfam database (https://www.ebi.ac.uk/interpro/entry/pfam) and NCBI-CDD (https://www.ncbi.nlm.nih.gov/Structure/bwrpsb/bwrpsb.cgi), were used to determine whether all candidate *PsWRKYs* contained the entire *WRKY* structural domain. ExPASy (https://web.expasy.org/protparam/) was used to calculate the amino acid length, molecular weight (MW), isoelectric point (pI), and instability index of all candidate PsWRKYs, whereas WoLF PSORT (https://wolfpsort.hgc.jp) was used to predict subcellular localisation [61, 62].

### Phylogenetic analysis and multiple sequence alignment of *PsWRKYs*

MEGA 11.0 software with the Cluster W tool was used for multi-protein sequence alignment analysis of WRKYs from *P. sibirica* and *A. thaliana*, and a phylogenetic evolutionary tree was constructed using default parameter values and the neighbour-joining method with 1000 bootstrap iterations [63]. Subsequently, the ITOLS v6.7.2 program (http://itol.embl.de) was used for phylogenetic tree visualization [64]. Classification of *PsWRKY* into relevant taxa based on a previously reported classification of the *A. thaliana WRKY* gene family.

### Conserved Motifs and Gene structure analysis of *PsWRKYs*

The TBtools v1.089 software was used to analyse and visualise the exon-intron structure of *PsWRKY*. The MEME v5.1.1 online program (https://meme-suite.org/meme/tools/meme) was used to predict the conserved motifs of WRKY proteins with the number of motifs set to 10 [65].

### Chromosomal localization, gene duplication, and covariance analysis of *PsWRKYs*

The *P. sibirica* genomic data annotation file was downloaded to determine chromosome length and gene location based on *PsWRKY* member IDs. TB tools were used to map the distribution of chromosomal localisation. MCScanX [66] was used to check for gene duplication events using default parameters. Dual systemic plots using TBtools for *P. sibirica* with *A. thaliana*, *P. salicina*, *P. mume*, and *P. persica WRKYs* interspecies covariance were analysed, and intraspecific gene duplicates of *P. sibirica* were analysed using Advanced Circos from TBtools. The Ka Ks_Calclator tool was used to calculate the frequency of synonymous mutation rate (Ks), frequency of non-synonymous mutation rate (Ka), and the ratio of nonsynonymous mutation rate to synonymous mutation rate (Ka/Ks) for *WRKY* duplicated genes in *P. sibirica*.

### Promoter *Cis*-acting element and GO annotation analysis of *PsWRKYs*

The PlantCARE database (https://bioinformatics.psb.ugent.be/webtools/plantcare/html/) was used to predict cis-acting elements of the 500 bp promoter sequence of *PsWRKYs* [67]. Eggnog 5.0 (http://eggnog5.embl.de/) was used for GO annotation and the results were visualized using WeGo 2.0 (https://wego.genomics.cn/).

### RNA isolation, and qRT-PCR validation

The primers were done using Primer Premier 5.0; their synthesis was entrusted to GENEWIZ (Suzhou, China), and 18 S rRNA served as a reference gene [68] (Table S2). Total RNA was extracted from the samples using an RNAprep Pure Plant Kit (Tiangen, Beijing, China), and cDNA was obtained using a FastKing RT Kit (Tiangen), according to the manufacturer’s instructions. The qRT-PCR was performed on a StepOne Real-Time PCR System (Applied Biosystems) using SuperReal PreMix Plus (SYBR Green, Tiangen). The PCR reaction program consisted of 95 °C for 30 s, followed by 40 cycles of 95 °C for 10 s, 60 °C for 30 s, 95 °C for 15 s, and 60 °C for 1 min, finishing with 95 °C for 15 s. The experiment was conducted three times and the relative expression level was determined using the 2^−∆∆CT^ method [69]. Statistical analysis was performed using SPSS software version 26.0. Bar graphs were created using GraphPad Prism 8.4.0 software.

## Electronic supplementary material

Below is the link to the electronic supplementary material.

**Additional file 1** Table S1. Detailed information of all identified *Prunus sibirica* WRKY proteins. Table S2. Primer pairs used for qRT-PCR analysis on the target sequences in *P. sibirica.* Table S3. Quantitative real-time PCR candidate gene information. Table S4. Mass concentrations in aseptic seedlings of *P. sibirica* total RNA. Figure S1. Cis-acting elements of the *PsWRKYs*. Figure S2. Phylogenetic tree of *WRKY* gene family from *P. sibirica*, *P. mume*, *P. persica*, and *P. armeniaca*

## Data Availability

The WRKY domain HMM (Hidden Markov Model) profile numbered PF03106 was extracted from the Pfam protein family database (https://www.ebi.ac.uk/interpro/entry/pfam/PF03106/). *P.sibirica* genome data were downloaded from the Rosaceae genome database (https://www.rosaceae.org/Analysis/10254124). The AtWRKY sequences were obtained from the Arabidopsis (*A. thaliana*) database (https://www.arabidopsis.org/). The datasets analysed during this study are included in this published article and its supplementary information files.

## Abbreviations

Ka: Non-synonymous substitution rate
Ks: Synonymous substitution rate
Ka/Ks: Ratio of the non-synonymous substitution rate to the synonymous substitution rate
qRT-PCR: Quantitative real-time PCR
GO: Gene ontology
