# Supplementary material for: Genome-wide identification and analysis of the WRKY gene family and low-temperature stress response in Prunus sibirica
Source: BMC Genomics. 2023 Jun 27;24:358. doi: 10.1186/s12864-023-09469-0 (PMC10294535; doi:10.1186/s12864-023-09469-0)
Supplement: Supplementary file 1 — Additional file 1 Table S1. Detailed information of all identified Prunus sibirica WRKY proteins. Table S2. Primer pairs used for qRT-PCR analysis on the target sequences in P. sibirica. Table S3. Quantitative real-time PCR candidate gene information. Table S4. Mass concentrations in aseptic seedlings of P. sibirica total RNA. Figure S1. Cis-acting elements of the PsWRKYs. Figure S2. Phylogenetic tree of WRKY gene family from P. sibirica, P. mume, P. persica, and P. armeniaca [file 12864_2023_9469_MOESM1_ESM.docx]

Table S1*.* Detailed information of all identified *Prunus sibirica* WRKY proteins

| Protein name | Sequence ID | Number of amino acids | Molecular weight  /Da | pI | Instability index | Aliphatic index | Grand average of hydropathicity | Chromosomal Location | Subcellular location prediction | Group |
| --- | --- | --- | --- | --- | --- | --- | --- | --- | --- | --- |
| PsWRKY1 | PaF106G0100000825.01 | 284 | 30910.93 | 9.91 | 48.58 | 65.6 | -0.58 | Chr1 | Nuclear | II-d |
| PsWRKY2 | PaF106G0100001343.01 | 388 | 42657.78 | 5.95 | 63.4 | 47.58 | -0.886 | Chr1 | Nuclear | II-c |
| PsWRKY3 | PaF106G0100002721.01 | 185 | 20880.43 | 9.56 | 44.73 | 54.81 | -0.821 | Chr1 | Nuclear | II-c |
| PsWRKY4 | PaF106G0100003357.01 | 515 | 55892.53 | 8.13 | 62.24 | 50.82 | -0.924 | Chr1 | Nuclear | I |
| PsWRKY5 | PaF106G0100003388.01 | 424 | 46980.96 | 10.33 | 55.69 | 60.8 | -0.764 | Chr1 | Nuclear | II-c |
| PsWRKY6 | PaF106G0100004627.01 | 325 | 36428.04 | 7.63 | 55.27 | 63.94 | -0.733 | Chr1 | Nuclear | II-a |
| PsWRKY7 | PaF106G0100004628.01 | 285 | 31577.44 | 8.38 | 51.29 | 75.65 | -0.599 | Chr1 | Nuclear | II-a |
| PsWRKY8 | PaF106G0100004783.01 | 163 | 18833.51 | 5.12 | 46.95 | 48.34 | -1.164 | Chr1 | Nuclear | II-c |
| PsWRKY9 | PaF106G0100005037.01 | 364 | 39656.95 | 9.32 | 54.08 | 59.23 | -0.573 | Chr1 | Nuclear | II-d |
| PsWRKY10 | PaF106G0100005329.01 | 345 | 37660.39 | 9.42 | 56.09 | 58.26 | -0.649 | Chr1 | Nuclear | II-d |
| PsWRKY11 | PaF106G0100006525.01 | 562 | 62075 | 5.21 | 52.62 | 58.54 | -0.963 | Chr1 | Nuclear | II-b |
| PsWRKY12 | PaF106G0200008520.01 | 244 | 27730.33 | 7.29 | 44.4 | 44.67 | -1.026 | Chr2 | Nuclear | II-c |
| PsWRKY13 | PaF106G0200009086.01 | 363 | 40001.12 | 7.16 | 54.03 | 48.9 | -0.837 | Chr2 | Nuclear | II-e |
| PsWRKY14 | PaF106G0200009090.01 | 221 | 24717.93 | 9.22 | 45.21 | 68.42 | -0.725 | Chr2 | Peroxisome | II-c |
| PsWRKY15 | PaF106G0200009172.01 | 358 | 39659.14 | 5.03 | 43.98 | 61.62 | -0.582 | Chr2 | Nuclear | III |
| PsWRKY16 | PaF106G0200009748.01 | 209 | 23893.9 | 8.9 | 46.54 | 61.1 | -0.904 | Chr2 | Peroxisome | II-c |
| PsWRKY17 | PaF106G0200010134.01 | 323 | 36428.42 | 5.7 | 47.03 | 56.13 | -0.853 | Chr2 | Nuclear | III |
| PsWRKY18 | PaF106G0200010574.01 | 296 | 32774.38 | 9.68 | 54.08 | 67.26 | -0.603 | Chr2 | Nuclear | II-e |
| PsWRKY19 | PaF106G0200010641.01 | 290 | 31551.62 | 5.02 | 57.96 | 51.52 | -0.74 | Chr2 | Nuclear | III |
| PsWRKY20 | PaF106G0300011395.01 | 505 | 55472.74 | 6.62 | 51 | 64.93 | -0.687 | Chr3 | Nuclear | II-b |
| PsWRKY21 | PaF106G0300011494.01 | 533 | 58223.38 | 8.18 | 62.79 | 56.75 | -0.892 | Chr3 | Nuclear | I |
| PsWRKY22 | PaF106G0300012051.01 | 652 | 71121.59 | 6.31 | 49.94 | 59.92 | -0.798 | Chr3 | Nuclear | II-b |
| PsWRKY23 | PaF106G0300012177.01 | 486 | 52923.2 | 5.9 | 41.99 | 63.29 | -0.903 | Chr3 | Nuclear | I |
| PsWRKY24 | PaF106G0300012509.01 | 363 | 41058.09 | 6.89 | 53.75 | 49.45 | -1.066 | Chr3 | Nuclear | II-c |
| PsWRKY25 | PaF106G0300013322.01 | 258 | 28060.17 | 5.13 | 54.12 | 55.19 | -0.696 | Chr3 | Nuclear | II-e |
| PsWRKY26 | PaF106G0300013492.01 | 272 | 30035.4 | 8.12 | 48.26 | 65.96 | -0.795 | Chr3 | Nuclear | II-a |
| PsWRKY27 | PaF106G0300014526.01 | 165 | 18526.84 | 9.3 | 64.88 | 53.21 | -0.681 | Chr3 | Nuclear | II-c |
| PsWRKY28 | PaF106G0300014596.01 | 611 | 66391.05 | 6.23 | 54.24 | 64.14 | -0.588 | Chr3 | Nuclear | II-b |
| PsWRKY29 | PaF106G0400015495.01 | 587 | 64119.61 | 6 | 50.75 | 54.92 | -0.831 | Chr4 | Nuclear | I |
| PsWRKY30 | PaF106G0400015665.01 | 536 | 58725.97 | 6.02 | 48.68 | 66.44 | -0.544 | Chr4 | Nuclear | II-b |
| PsWRKY31 | PaF106G0400017105.01 | 527 | 57094.68 | 6.06 | 56.09 | 54.52 | -0.761 | Chr4 | Nuclear | II-e |
| PsWRKY32 | PaF106G0400017434.01 | 340 | 37588.33 | 6.55 | 55.43 | 48.53 | -0.941 | Chr4 | Nuclear | II-c |
| PsWRKY33 | PaF106G0400017534.01 | 342 | 38516.69 | 9.16 | 53.39 | 50.23 | -0.691 | Chr4 | Nuclear | II-e |
| PsWRKY34 | PaF106G0400018245.01 | 617 | 67027.07 | 7.34 | 46.93 | 60.16 | -0.761 | Chr4 | Nuclear | II-b |
| PsWRKY35 | PaF106G0500019357.01 | 330 | 36942.17 | 9.68 | 54.35 | 72.7 | -0.623 | Chr5 | Nuclear | II-d |
| PsWRKY36 | PaF106G0500019756.01 | 314 | 35487.83 | 5.18 | 60.23 | 70.83 | -0.593 | Chr5 | Nuclear | II-e |
| PsWRKY37 | PaF106G0500019874.01 | 248 | 28031.79 | 5.12 | 64.54 | 47.22 | -0.866 | Chr5 | Nuclear | III |
| PsWRKY38 | PaF106G0500020691.01 | 683 | 73580.87 | 6.95 | 49.02 | 55.94 | -0.811 | Chr5 | Nuclear | II-b |
| PsWRKY39 | PaF106G0500020010.01 | 205 | 23126.62 | 4.78 | 31.07 | 72.73 | -0.768 | Chr5 | Chloroplast | I |
| PsWRKY40 | PaF106G0600021797.01 | 697 | 75002.92 | 5.61 | 52.3 | 53.49 | -0.776 | Chr6 | Nuclear | I |
| PsWRKY41 | PaF106G0600021917.01 | 590 | 64692.37 | 6.58 | 52.5 | 45.15 | -0.945 | Chr6 | Nuclear | I |
| PsWRKY42 | PaF106G0600023566.01 | 196 | 21998.11 | 6.21 | 56.69 | 42.19 | -0.989 | Chr6 | Nuclear | II-c |
| PsWRKY43 | PaF106G0600024291.01 | 326 | 35586.26 | 9.6 | 41.21 | 67.33 | -0.54 | Chr6 | Nuclear | II-d |
| PsWRKY44 | PaF106G0600024432.01 | 479 | 52136.65 | 8.91 | 49.61 | 55.55 | -0.92 | Chr6 | Nuclear | I |
| PsWRKY45 | PaF106G0600024597.01 | 252 | 28590.67 | 9.27 | 47.6 | 62.34 | -0.775 | Chr6 | Peroxisome | II-c |
| PsWRKY46 | PaF106G0600024936.01 | 536 | 59540.37 | 7.17 | 62.54 | 48.21 | -0.94 | Chr6 | Nuclear | I |
| PsWRKY47 | PaF106G0600025042.01 | 354 | 39732.74 | 5.65 | 53.66 | 57.37 | -0.781 | Chr6 | Nuclear | III |
| PsWRKY48 | PaF106G0600025043.01 | 337 | 38056.18 | 5.5 | 45.51 | 61.6 | -0.698 | Chr6 | Nuclear | III |
| PsWRKY49 | PaF106G0600025044.01 | 336 | 37434.25 | 5.81 | 49.6 | 61.22 | -0.708 | Chr6 | Nuclear | III |
| PsWRKY50 | PaF106G0600025642.01 | 355 | 40054.93 | 9.68 | 50.11 | 69.18 | -0.763 | Chr6 | Nuclear | II-d |
| PsWRKY51 | PaF106G0600025823.01 | 512 | 56847.87 | 6.53 | 52.82 | 59.22 | -0.818 | Chr6 | Nuclear | I |
| PsWRKY52 | PaF106G0700026001.01 | 533 | 58804.27 | 5.54 | 57.32 | 66.02 | -0.834 | Chr7 | Nuclear | I |
| PsWRKY53 | PaF106G0800029338.01 | 262 | 29836.13 | 5.29 | 67.04 | 58.36 | -1.011 | Chr8 | Nuclear | II-d |
| PsWRKY54 | PaF106G0800029773.01 | 355 | 40061.3 | 9.61 | 58.09 | 62.59 | -0.85 | Chr8 | Nuclear | II-d |
| PsWRKY55 | PaF106G0800031110.01 | 291 | 32971.63 | 8.91 | 44.92 | 79.07 | -0.387 | Chr8 | Nuclear | II-c |

Table S2. Primer pairs used for qRT-PCR analysis on the target sequences in *P. sibirica*

| PsWRKY | Primer pairs | |
| --- | --- | --- |
|  | Forward primer (5'-3') | Reverse primer (5'-3') |
| PsWRKY1 | TCGTCGGGTTCTCAAGGAT | TCGGTGATTTCTCGGCAGT |
| PsWRKY4 | GGCAGAAAGTCGTCAAAG | GTTGTTGGCTGCGTAGTG |
| PsWRKY6 | TATTCCTTTCCACCCTGCTAG | TGCTGCCTCTTGTTTCACC |
| PsWRKY9 | TCTTCCTCATCGAACCCTG | GGAGGCGGAGATGGAGT |
| PsWRKY10 | CCAGCAGCAAACTAACCAG | TGTGAATACGAGGGATGAGAAG |
| PsWRKY12 | CTTAGGGAGCCAAGATTC | TTCTTCACCCGACAGTTAC |
| PsWRKY13 | AAAGGGCAGTCCTATGGTC | ACAAAGAAATCGTCGCTCAC |
| PsWRKY14 | TAATGTTGGTGCCTCTGCT | CTGGCTTTCTCATCTTACCC |
| PsWRKY18 | AACAAGTGGAGCGGAGTAC | CGTTGGACGAGGGTGATT |
| PsWRKY21 | ATGGCGGTGAGAACAAGTC | AAAGAAGCCAGGCGAGTTAAG |
| PsWRKY27 | TGGCTACAGATGGAGAAAG | GAGTTGGGCGAGGTATGAG |
| PsWRKY36 | TAGCAGGAAGAATCAGAGC | TGCCCATATTTACGCCAC |
| PsWRKY38 | AATCATTGGCAGCAACAGT | GCGTTTGGAGGTATGGAGAAG |
| PsWRKY42 | ATGGGTCATAGGGTTGCTT | TTGGATTTGGGCTGTTCTT |
| PsWRKY44 | ATCAGTCGAGGCTGGTGTT | TATTGGCTGTGGACGGTAT |
| PsWRKY51 | TGCCTCAGCCTAATCGT | CCAGACCTTCAGCCCTC |
| PsWRKY52 | GACATGCCTGTTCCAAAG | TCACCAGTCAATTCTCCC |
| PsWRKY54 | GGAGACGATGGGAGTGT | TTGGCTTCTGTCCATACTT |
| PsWRKY55 | ACCAAGCCACGAGAACACT | CCAACAGCATACCGAATAG |
| PsWRKY15 | CTGGAACAGGGCTAGATGG | TTTGAACTTGCTTGGTGGC |
| PsWRKY17 | TTTTGGCGAGGTTTATC | CCTGCACCTTCCTACTCT |
| PsWRKY49 | TCAAATAGGGAAAGGGTG | CCTCTGCCGACTCAACAT |
| PsWRKY5 | GCTTGTTGCGGACTGCTTAC | GTATCTCGGGCGGTGGTTT |
| 18SrRNA | AAACGGCTACCACATCCA | CACCAGACTTGCCCTCCA |

Table S3. Quantitative real-time PCR candidate gene information

| Gene ID | Sequence ID | Gene Information |
| --- | --- | --- |
| *PsWRKY1* | PaF106G0100000825.01 | Contains LTR cis-acting elements |
| *PsWRKY4* | PaF106G0100003357.01 | Contains LTR cis-acting elements; response to stimuli (GO:0050896) |
| *PsWRKY5* | PaF106G0100003388.01 | reproductive process (GO:0022414); reproduction (GO:0000003); Homologs of the cold resistance gene *PmWRKY57* in *Prunus mume* [16] |
| *PsWRKY6* | PaF106G0100004627.01 | regulation of response to stimulus (GO:0048583) |
| *PsWRKY9* | PaF106G0100005037.01 | Contains cis-acting regulatory element involved in the MeJA-responsiveness |
| *PsWRKY10* | PaF106G0100005329.01 | response to external stimulus (GO:0009605); |
| *PsWRKY12* | PaF106G0200008520.01 | Homologs of the cold resistance gene *PmWRKY57* in *Prunus mume*[16] |
| *PsWRKY13* | PaF106G0200009086.01 | Contains LTR cis-acting elements; developmental process (GO:0032502) |
| *PsWRKY14* | PaF106G0200009090.01 | Contains a variety of cis-acting elements associated with plant growth and development |
| *PsWRKY15* | PaF106G0200009172.01 | Homologs of the frost resistance gene *PARG18453* in *Prunus armeniaca* ; developmental process (GO:0032502) |
| *PsWRKY17* | PaF106G0200010134.01 | regulation of response to stimulus (GO:0048583); response to external stimulus (GO:0009605); Homologs of the frost resistance gene *PARG18453* in *Prunus armeniaca* |
| *PsWRKY18* | PaF106G0200010574.01 | response to external stimulus (GO:0009605) |
| *PsWRKY21* | PaF106G0300011494.01 | Contains LTR cis-acting elements |
| *PsWRKY27* | PaF106G0300014526.01 | Contains LTR cis-acting elements; response to external stimulus (GO:0009605) |
| *PsWRKY36* | PaF106G0500019756.01 | Contains LTR cis-acting elements |
| *PsWRKY38* | PaF106G0500020691.01 | Contains LTR cis-acting elements |
| *PsWRKY42* | PaF106G0600023566.01 | response to external stimulus (GO:0009605) |
| *PsWRKY44* | PaF106G0600024432.01 | reproductive process (GO:0022414); reproduction (GO:0000003) |
| *PsWRKY49* | PaF106G0600025044.01 | regulation of response to stimulus (GO:0048583); response to external stimulus (GO:0009605); Homologs of the frost resistance gene *PARG18453* in *Prunus armeniaca* |
| *PsWRKY51* | PaF106G0600025823.01 | reproductive process (GO:0022414); reproduction (GO:0000003); developmental process (GO:0032502) |
| *PsWRKY52* | PaF106G0700026001.01 | Contains LTR cis-acting elements |
| *PsWRKY54* | PaF106G0800029773.01 | Contains LTR cis-acting elements |
| *PsWRKY55* | PaF106G0800031110.01 | Contains LTR cis-acting elements |

Note: LTR cis-acting element involved in low-temperature responsiveness

Table S4. Mass concentrations in aseptic seedlings of *P. sibirica* total RNA

| Type | RNA concentration（ng.μL^-1^） | RNA integrity number |
| --- | --- | --- |
| 0 h | 786 | 9.1 |
| 15 min | 987 | 7.2 |
| 30 min | 1408 | 8.3 |
| 1 h | 1186 | 9.1 |
| 2 h | 1097 | 7.3 |
| petal | 628 | 8.6 |
| pistil | 969 | 8.6 |
| root | 1178 | 8.7 |
| stem | 1103 | 8.9 |
| leaf | 927 | 7.1 |


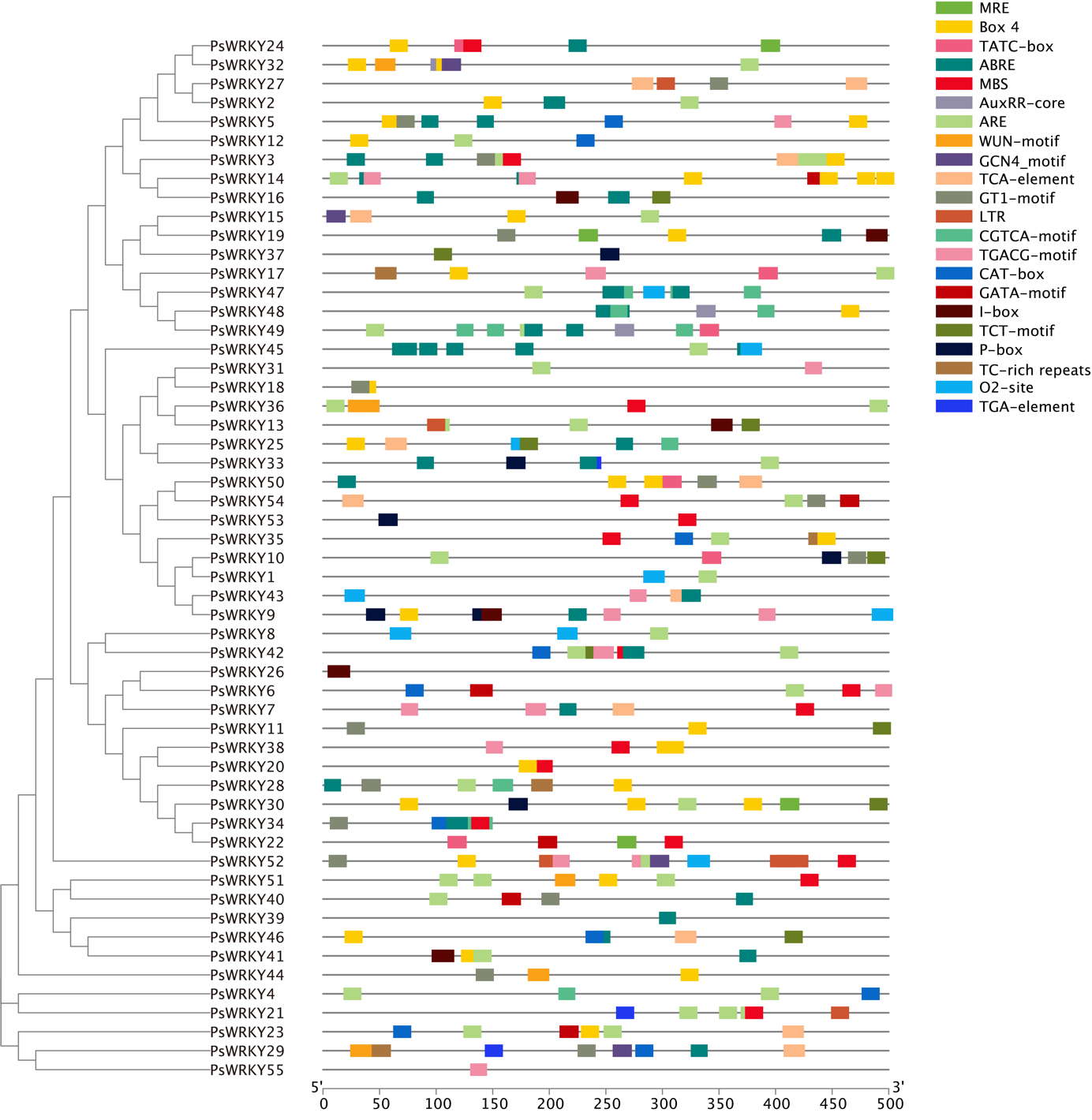


Figure S1. Cis-acting elements of the *PsWRKYs*

*
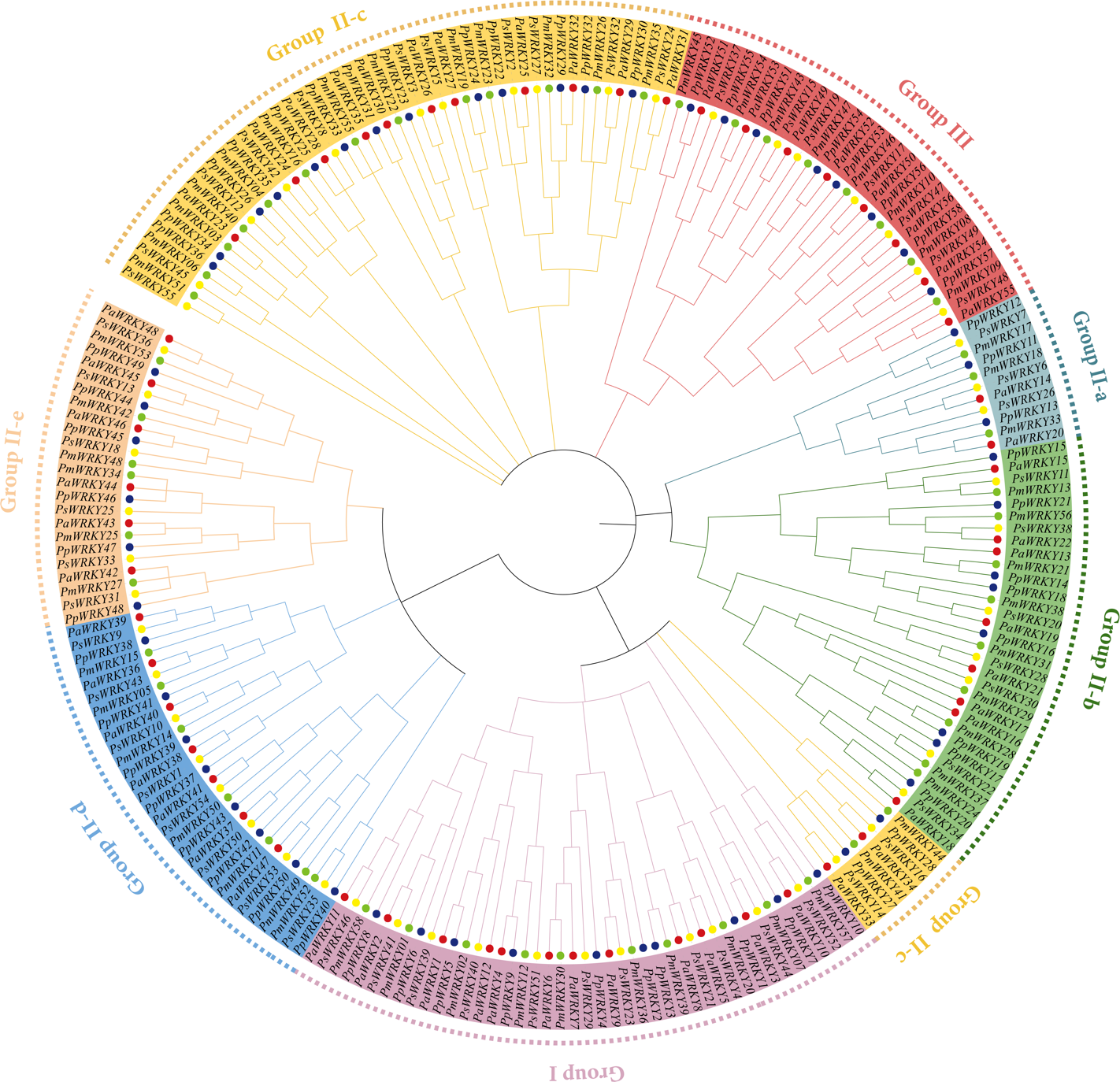
*

Figure S2. Phylogenetic tree of *WRKY* gene family from *P. sibirica*, *P. mume*, *P. persica*, and *P. armeniaca*.

Subgroups of *WRKY* gene family are highlighted with different colors. *P. sibirica*, *P. mume*, *P. persica*, and *P. armeniaca* are marked as yellow, green, blue, and red circles, respectively.
